# Unravelling the genetic basis and regulation networks related to fibre quality improvement using chromosome segment substitution lines in cotton

**DOI:** 10.1111/pbi.14436

**Published:** 2024-07-24

**Authors:** Guoan Qi, Zhanfeng Si, Lisha Xuan, Zegang Han, Yan Hu, Lei Fang, Fan Dai, Tianzhen Zhang

**Affiliations:** ^1^ Hainan Institute of Zhejiang University, Yazhou Bay Science and Technology City Sanya Hainan China; ^2^ The Advanced Seed Institute, College of Agriculture and Biotechnology, Zhejiang University Hangzhou Zhejiang China

**Keywords:** CSSL, regulation network, substitution detection, large‐scale transcriptional profiling, fibre quality

## Abstract

The elucidation of genetic architecture and molecular regulatory networks underlying complex traits remains a significant challenge in life science, largely due to the substantial background effects that arise from epistasis and gene–environment interactions. The chromosome segment substitution line (CSSL) is an ideal material for genetic and molecular dissection of complex traits due to its near‐isogenic properties; yet a comprehensive analysis, from the basic identification of substitution segments to advanced regulatory network, is still insufficient. Here, we developed two cotton CSSL populations on the *Gossypium hirsutum* background, representing wide adaptation and high lint yield, with introgression from *G. barbadense*, representing superior fibre quality. We sequenced 99 CSSLs that demonstrated significant differences from *G. hirsutum* in fibre, and characterized 836 dynamic fibre transcriptomes in three crucial developmental stages. We developed a workflow for precise resolution of chromosomal substitution segments; the genome sequencing revealed substitutions collectively representing 87.25% of the *G. barbadense* genome. Together, the genomic and transcriptomic survey identified 18 novel fibre‐quality‐related quantitative trait loci with high genetic contributions and the comprehensive landscape of fibre development regulation. Furthermore, analysis determined unique *cis*‐expression patterns in CSSLs to be the driving force for fibre quality alteration; building upon this, the co‐expression regulatory network revealed biological relationships among the noted pathways and accurately described the molecular interactions of *GhHOX3*, *GhRDL1* and *GhEXPA1* during fibre elongation, along with reliable predictions for their interactions with *GhTBA8A5*. Our study will enhance more strategic employment of CSSL in crop molecular biology and breeding programmes.

## Introduction

Most of the traits important in agronomy are so‐called complex traits featuring polygenic inheritance and complex molecular regulation. At base, complex traits result from genetic masking of multiple QTLs from one another as a result of co‐segregation and interaction with the environment; combined with the continuous loss of genetic diversity due to long‐term artificial selection during plant domestication, this has greatly limited QTL mapping and genetic breeding research in crops (Gepts *et al*., 2012; Mackay, 2001). Furthermore, while significant advances have been made in the genomic and transcriptomic understanding of complex traits, most studies have focused on the mechanisms of individual QTLs or genes with major genetic effects. Compared to the conventional reductionist approach that characterizes the relationship between genes and phenotypes, a holistic perspective grounded in network analysis evidently aligns more closely with and aptly describes the intricacies inherent in biological outcomes. However, the comprehensive interpretation of gene interactions and cellular networks in complex genomes remains a major scientific challenge (Costanzo *et al*., 2011; Guimerà and Nunes Amaral, 2005; Saint‐Antoine and Singh, 2020).

Chromosome segment substitution lines (CSSLs) are characterized by genomes that are identical except for one or a handful of genomic segments introgressed from distant or wild species. Consequently, phenotypic variation among CSSLs can be directly attributed to the foreign segments, making them ideal for dissecting the mechanisms of genetic and transcriptional regulation of complex quantitative traits (Balakrishnan *et al*., 2019). Recently, CSSL populations have been widely utilized in QTL detection and gene interaction studies in important crops such as tomato, rice, maize, cotton and soybean (Fonceka *et al*., 2012; Li *et al*., 2022; Lopez‐Zuniga *et al*., 2019; Schauer *et al*., 2006; Wang *et al*., 2013; Xi *et al*., 2006). Importantly, the identical and stable genetic background in CSSLs also precludes potential interference from genome‐wide epistatic interactions; with the addition of omics methods like transcriptomics and metabolomics, CSSLs constitute a powerful genetic panel for studying molecular and metabolic interactions and networks (Coombes *et al*., 2023; Ma *et al*., 2022; Schauer *et al*., 2006; Szymański *et al*., 2020). However, most studies in CSSLs to date lack high‐precision identification of the introgression segments, barely method or software for introgression detection is available, and only limited attention has been paid to the unique *cis*‐ and *trans*‐regulatory patterns in CSSLs. How traits are affected by introgression segments, including both impact and causality, and the transcriptomic and metabolic changes induced by such segments are still largely unknown.

Cotton is the most vital natural fibre resource with worldwide economic value. Over the last 100 years, domestication of the major tetraploid cotton species *Gossypium hirsutum* has led to tremendous yield gains; at the same time, continuous selection has narrowed the genetic basis of disease resistance and fibre quality. Such genetic bottlenecks pose a great challenge for the genetic breeding of cotton, especially in the context of ever‐increasing demand for improvements in both lint yield and fibre quality (Wendel *et al*., 1989). In contrast to the field characteristics of *G. hirsutum*, another tetraploid cotton, *G. barbadense*, is known for its extremely high‐quality fibre. Introduction of genomic segments from *G. barbadense* into *G. hirsutum* is thus an ideal way to improve fibre quality while ensuring high lint yield. Novel variations brought by the foreign segments not only bring genetic diversity into the adaptive genome, but also disrupt the tight genetic linkage of traits. Recently, more and more cotton CSSL and backcross inbred line (BIL) populations have been constructed, and QTLs related to fibre quality and lint yield have been identified using either simple sequence repeat (SSR), kmer‐based or emerging single nucleotide polymorphism (SNP) markers (Guo *et al*., 2013; Li *et al*., 2019, 2022; Ma *et al*., 2022; Wang *et al*., 2012a, 2012b; Zhai *et al*., 2016). Nevertheless, few studies to date have explored the regulation of fibre in its critical developmental periods. A comprehensive understanding of relevant molecular pathways and the interactive regulatory network that governs fibre development is therefore still lacking.

In this study, we developed and re‐sequenced 99 CSSLs harbouring genome‐wide introgressions using *Gossypium hirsutum* acc. TM‐1 as the recurrent parent and *Gossypium barbadense* acc. 3–79 and cv. Hai7124 as the donor parent. Additionally, a total of 836 dynamic transcriptomes were characterized at three key fibre developmental stages: 0, 10 and 20 days post‐anthesis (DPA). We developed an innovative and effective pipeline based on high‐density SNP markers, and proposed a scripting utility Substitution Segment Detection (SSD) for identifying introgression segments. The detected segments were further incorporated into the TM‐1 genome for transcriptomic analysis to study the expression patterns of the introgressed genes. Integrating genomic and transcriptomic analysis with phenotype profiling on three major fibre quality traits, fibre length (FL), fibre strength (FS) and fibre micronaire (FM), we dissected the genetic basis of fibre quality improvement in the CSSL population, which yielded several novel QTLs with high genetic contributions. Finally, based on the large‐scale transcriptional data, we examined differentially expressed pathways related to fibre quality and regulatory kernels among *cis*‐ and *trans*‐expression architectures.

## Results

### Construction and phenotypic evaluation of CSSLs

The cotton CSSL population constructed in this study used TM‐1, the *G. hirsutum* genetic standard line, as the recurrent parental line, and *G. barbadense* cv. Hai7124 and acc. 3–79 as donor parental lines. Crossing of TM‐1 with a donor parental line was followed by advanced backcrossing, after which marker‐assisted selection (MAS) was applied to the backcross population to identify and retain plants with foreign segments located at specific genomic regions. The selected cotton plants in BC_5‐6_ were further self‐fertilized for four generations, resulting in the development of two sets of CSSLs: 174 CSSLs with Hai7124 introgression (Hai7124_CSSL) (Wang *et al*., 2008, 2012b) and 166 CSSLs with 3–79 introgression (3–79_CSSL) (Figure S1A, Appendix S1). The CSSLs exhibit wide‐ranging variabilities in plant architecture, fibre quality and lint yield (Tables S1 and S2). Based on our field data, 99 CSSLs that differed significantly from TM‐1 (the recurrent parent) in either yield or fibre qualities were selected for this research. Three important fibre quality traits, FL, FS and FM, were investigated in this CSSL population. Fibre quality was normally distributed throughout the population and showed significant differences compared to the recurrent parent TM‐1, as expected (Figure S1B and Table S3). For example, the FL of TM‐1 is 29.16 mm, while in the CSSL population, it ranges up to 31.47 mm in CSSL‐2649, an extension of 7.9%, and down to only 23.5 mm in CSSL‐2611, a shortening of 19.41%. FS and FM also exhibited considerable phenotypic variation in the CSSL population, with respective value ranges of −16.15% to 21.82% and −25.45% to 14.75% compared to the recurrent parent. As the CSSLs are near‐isogenic lines on the TM‐1 background, this phenotypic variation can be directly attributed to the foreign substitution segments, making this population ideal for studying the mechanisms of genetic and transcriptional regulation in fibre quality establishment (Cao *et al*., 2014; Ecke *et al*., 2015).

### Precision identification of chromosomal substitution segments in CSSLs

**Figure 1:**
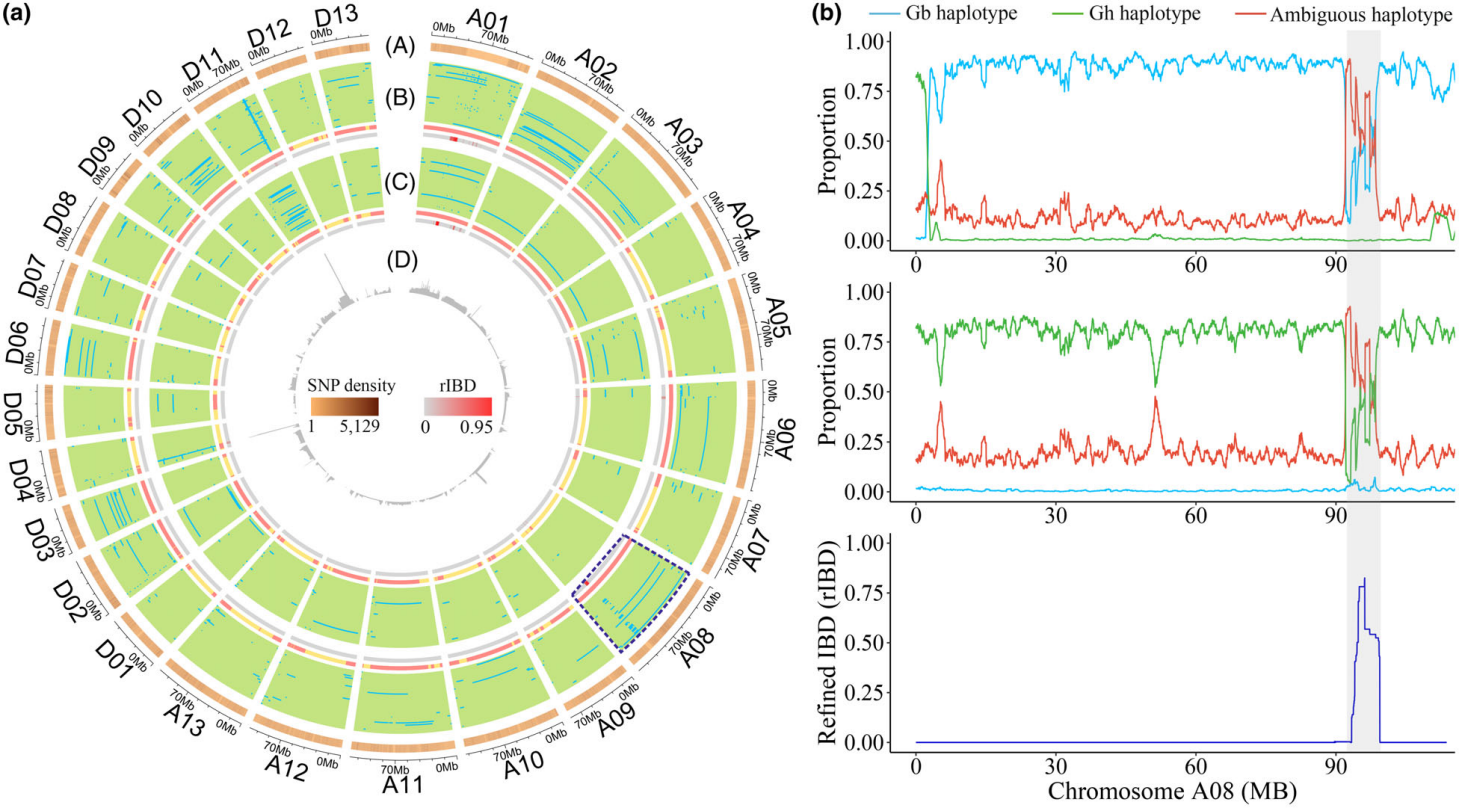
Substitution segment detection in the cotton CSSL population. (a) Overview of chromosomal substitution segments in 99 CSSLs. (A) Whole genome SNP density, plus overview of substitution segments in (B) 3–79 and (C) Hai7124 CSSLs. The first sub‐track in track (B) and (C) shows the substitution segments in each CSSL, with green representing the adaptive genomic background from the recurrent parent TM‐1 and blue representing the foreign segments introgressed from the donor parent. The second narrow sub‐track shows the combined substitution segments in all CSSLs, with light red indicating that at least one line carries a substitution segment in the corresponding genomic region, and light yellow indicating that all CSSLs retain the TM‐1 sequence in the region. The last sub‐track depicts the natural introgression segments shifted from recurrent parent TM‐1 to donor parent 3–79 or Hai7124, based on the rIBD index. (D) Substitution depth across the genome. (b) Key parameters in the detection of substitution segments in chromosome A08 of CSSL‐2668 (upper) and CSSL‐2669 (middle). The natural introgression segment shifts from donor parent 3–79 to recurrent line TM‐1 (bottom) are highlighted in grey.

We sequenced the whole genomes of 99 CSSLs with an average ~5.74‐fold depth. Mapping clean reads against the TM‐1 reference genome (Hu *et al*., 2019) revealed 19 444 373 high‐quality SNP markers in the CSSL population. Based on genotypic comparison of each CSSL with its parent lines, we determined the source parent of every allele and developed a workflow for precise resolution of chromosomal substitution segments (Figure S2, see Appendix S1 for more details). A total of 735 genomic segments transferred from *G. barbadense* were detected by SSD. The longest substitution segment of 115.73 Mb was found in CSSL‐2668, covering most of chromosome (chr.) A08, and the shortest of 0.13 Mb was found in CSSL‐2637 (Figure 1a, Table S4). The number of substitution segments carried in each CSSL mainly ranged from four to 10, with the summed size of segments mainly ranging from 21.42 Mb to 108.91 Mb (values are the first and third quartiles) (Figure S3). Across the selected lines, the foreign segments achieve a whole‐genome level of substitution, collectively representing about 87.25% of the *G. barbadense* genome; notably, these segments were uniformly distributed without sub‐genome or chromosome bias (Figures S4 and S5, Table S5).

To further demonstrate the sensitivity and precision of substitution segment detected in homologous regions that commonly present between *G. hirsutum* and *G. barbadense*, we performed refined IBD (rIBD) analysis using a prebuilt genetic panel of cotton cultivars (Fang *et al*., 2021) to detect the natural introgression shift from *G. hirsutum* TM‐1 to *G. barbadense* acc. 3–79 and cv. Hai7124 (the third sub‐track in Figure 1a‐B,C). Genomic regions with higher rIBD values are indicated to be homologous in the two parental lines. For instance, we found a typical natural introgression ranging from 91.96 to 98.58 Mb on chr. A08 (rIBD >0.1, Figure 1b, bottom panel) which had high similarity in the two parental lines; consequently, many alleles in this region in CSSL (~63.12% on average for CSSL‐2668 and CSSL‐2669) are of ambiguous source (Figure 1b). In CSSL‐2668 (Figure 1b, top panel), we can find out very few alleles (<1%) in this region to be from the recurrent line TM‐1 (recurrent lineage‐specific alleles, LSA, *i.e*. Gh haplotype), whereas most other alleles, except ambiguous alleles, were determined to be of donor parent origin (donor LSA, *i.e*. Gb haplotype); thus, this region in CSSL‐2668 was clearly inherited from the donor parent 3–79. In CSSL‐2669, the situation was just the opposite (Figure 1b, middle panel): with most alleles with ascertainable parent line being of TM‐1 origin and barely no alleles from *G. barbadense*, this region in CSSL‐2669 is assigned as belonging to the recurrent genome. This example illustrates how our method can clearly distinguish whether a region is a foreign substitution segment, even for homologous genomic regions.

### Genetic basis of fibre improvement in CSSL population

**Figure 2:**
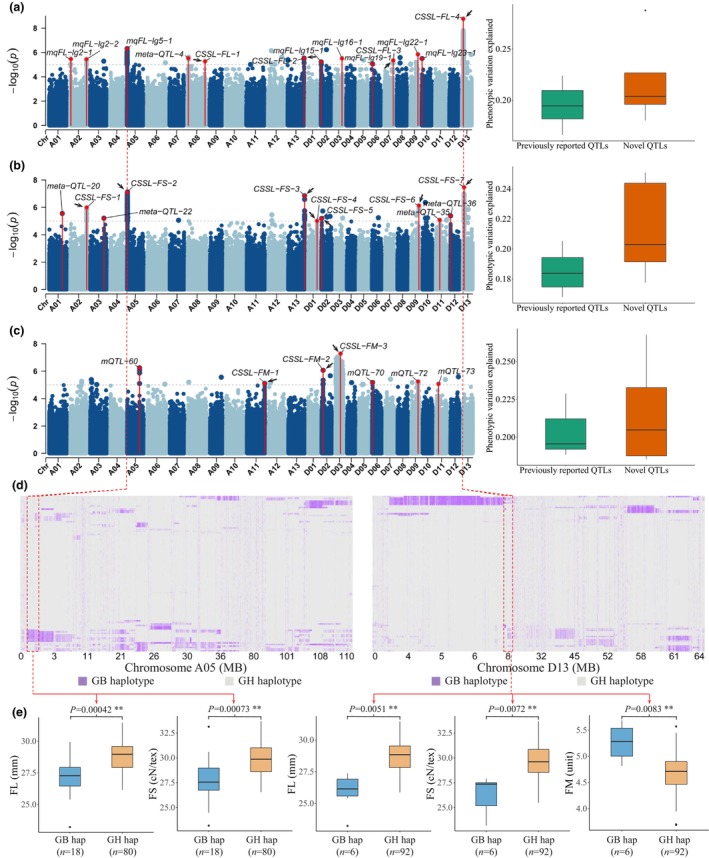
Genetic impact of foreign substitution segments on fibre quality in the CSSL population. Manhattan plot and phenotypic variation explained for potential QTLs of (a) FL, (b) FS and (c) FM‐GWAS. QTLs detected with steady signal peaks or jointly discovered in previous studies are marked with red signal lines; QTL labels are tagged near the significant variants. Novel QTLs discovered in this study are tagged with black arrows. (d) Haplotype heat maps for two exampled QTL located at chromosome A05 (left) and D13 (right). The genotype is coded according to whether it is introgressed from GB. (e) Phenotype analysis showed significant differences in fibre quality performance under different A05 QTL haplotypes (left two panels) and D05 QTL haplotypes (right three panels).

The phenotype of three important fibre quality traits, FL, FS and FM, were measured for CSSL population. Based on the phenotype profiling and 3 263 105 SNP markers with minor allele frequency >0.05 and missing rate <0.2, we conducted genome‐wide association studies (GWAS) to screening the potential QTLs. We identified 26 independent genomic regions significantly associated with FL, including nine QTLs reported in previous studies (Brown *et al*., 2019; Chandnani *et al*., 2018; Jia *et al*., 2018; Keerio *et al*., 2018; Lacape *et al*., 2005, 2009; Li *et al*., 2016, 2019; Liu *et al*., 2018; Ma *et al*., 2018, 2022; Shi *et al*., 2020; Tan *et al*., 2018), and four novel QTLs on chr. A08, A13, D07 and D13 (Figure 2a, Table S6). Notably, the novel QTLs were associated with higher phenotypic variation: The average phenotypic variation explained (PVE) by novel QTLs was 21.89%, higher than the 19.30% of previously reported QTLs (19.30%). Most remarkably, a newly found QTL named *CSSL‐FL‐4* located at chr. D13 with additive effect −2.11 (mm) was able to explain up to 28.78% of FL phenotypic variation (Figure S6). For FS, we identified 23 independent genomic regions significantly associated with FS, which included four QTLs previously reported (Diouf *et al*., 2018; Huang *et al*., 2017; Jia *et al*., 2018; Keerio *et al*., 2018; Liu *et al*., 2018; Tan *et al*., 2018) and seven QTLs located on chr. A02, A05, A13, D01, D02, D09 and D13 are newly identified in this study (Figure 2b, Table S7). The average PVE of the novel FS‐QTLs was 21.45%, with three on chr. A05, A13 and D13 explaining particularly high proportions of phenotypic variation at 24.59%, 25.09% and 24.18%, respectively. Meanwhile, the average PVE for the previously reported FS‐QTLs was 18.52%. In the GWAS for FM, 23 independent genomic regions were found to be significantly associated, in which six convincing QTLs were supported by continuous signal peaks, with four having been reported in previous studies (Figure 2c, Table S8) (Diouf *et al*., 2018; Jia *et al*., 2018; Li *et al*., 2016; Liu *et al*., 2018; Tan *et al*., 2018). As with other traits, the novel FM‐QTLs identified in this study were also able to explain a higher proportion of phenotypic variation.

The potential genetic impacts of foreign introgressions were further demonstrated by haplotype analysis of the QTLs located at chr. A05 (Figure 2d, left panel) and chr. D13 (Figure 2d, right panel). In lines with better fibre quality, elite haplotypes were introgressed from *G. barbadense*, while in those with poorer fibre performance, the corresponding elite haplotypes were present in *G. hirsutum*. For the QTL region at chr. A05, we found that 18 CSSLs with effective introgressions have worse performance in FL and FS; and six CSSLs with GB haplotypes in QTL regions at chr. D13 also have significantly poor FL, FS and FM (Figure 2e). Haplotype analysis of another striking GWAS signal on chr. D03, which explain 26.77% of FM variation, revealed chromosomal level of introgression in five of the 99 CSSLs; all of these lines demonstrated much lower FM values ranging from 3.69 to 4.31 (unit), which is generally considered to be the optimal FM interval, compared to other CSSL with TM‐1 background on chr. D03 (Figure S7). These results indicate sufficient introgression of important variation that influencing the fibre quality, which forms the genetic basis for the observed differences in fibre performance within the CSSL population.

### Effects of exotic genomic segments on transcriptomes of the CSSL population

With a total of 19 444 373 genomic variants, the genome‐wide substitution of TM‐1 in our CSSL population brought abundant new alleles; of those, 13 504 713 variants (69.45%) were found to have the *G. barbadense* haplotype in at least one CSSL. Functional annotation of variants in junction regions revealed that genetic recombination tended to occur in gene body, upstream and other genomic regions with potential biological functions, while a much lower proportion of the variants were distributed in intergenic regions (46.61% compared to 73.19% for all variants, Figure S8). This biased insertion may result from our phenotypic selection for agronomic characters. The widespread new variants and their distributional preference in functional regions supports that the introduction of foreign segments influenced the genetic and transcriptomic basis of fibre quality improvement in the CSSL population.

**Figure 3:**
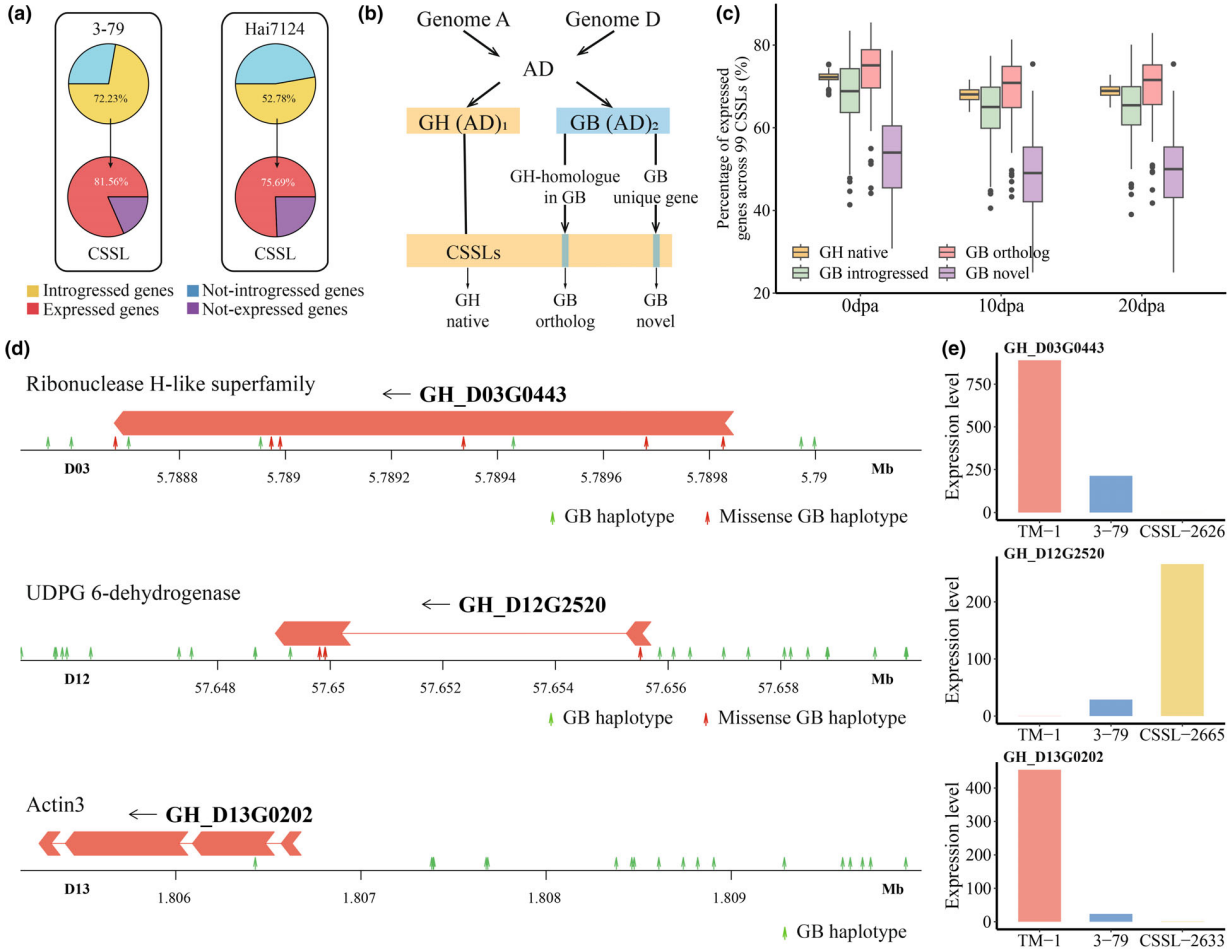
Expression patterns of exogenous substituted genes and transcriptomic impact of foreign haplotypes in the CSSL population. (a) Proportions of 3–79 and Hai7124 genes introduced into the 99 CSSLs and their expression capability after introgression. (b) Definition of gene sets according to their sources. (c) Expression capacity of native and foreign genes in ovule/fibre across 99 CSSL during three developmental stages. (d) Novel variations brought by foreign segments in affected genes. Red area represents the coding sequence (CDS) for the gene. (e) Expression levels of affected genes in a representative CSSL and its parent lines.

To investigate the expression status of *G. barbadense* genes within the *G. hirsutum* genome, we initially performed expression quantification of fibres at different developmental stages using the CSSL ‘pseudo‐genome’ as the reference genome (Appendix S1, Figure S9). Across the CSSL population, most (72.23%) 3–79 genes and more than half (52.78%) of Hai7124 genes were detected; transcriptome data from three key fibre development stages revealed most of these genes to be capable of expression (normalized count >1) in the new genetic environment (Figure 3a). We further classified the introgressed genes into those with homologues in TM‐1 (GB orthologue) and those without (GB novel), and used the native genes in TM‐1 (GH native) as a control to study their expression ability (Figure 3b). This revealed the GB orthologues to have expression capacity similar to GH native genes, with 73.08%, 69.02% and 69.62% of orthologues being expressed on average in the three development stages, compared to the 72.19%, 67.94% and 68.82% of native TM‐1 genes. Meanwhile. GB novel genes, which have no counterparts in *G. hirsutum*, showed obvious expression incompatibility with averaged 51.48%, 48.81% and 48.43% of genes respectively being expressed at the stages examined (Figure 3c, Table S9). This phenomenon is consistent with a previous study of wheat substitution lines, in which 44.1% of wheat orthologue and only 9.46% of novel genes were expressed (Coombes *et al*., 2023). Notably, GH native genes exhibited comparable expression capacities across the CSSL population at each developmental stage, whereas the introgression genes demonstrated significant expression variation among different CSSLs. However, differences in the overall expression capacity of introgressed genes seem to have little connection with fibre quality (Figure S10).

In order to compare the effects of the exotic haplotypes on gene expression from a unified perspective, we also quantified gene expression levels in CSSLs and their parental lines (TM‐1, 3–79 and Hai7124) using TM‐1 as the reference genome. A total of 23 384 genes that either contain or are proximal to foreign haplotypes were found to be differentially expressed in the CSSLs compared with their recurrent parent TM‐1, with each gene being affected by 35 exotic haplotypes on average. Especially, 311 genes showed magnitude changes (absolute log fold‐change >10) due to the impact of foreign haplotypes on upstream and downstream regulatory elements, as well as direct changes of protein sequence (Table S10). For example, *GH_D03G0443* (Ribonuclease H‐like superfamily member) expression in 10 DPA fibre was abolished in CSSL‐2626, in contrast to its extremely active transcription in TM‐1 (over 1577 normalized counts, NC hereafter) (Figure 3d). Functional annotation showed the foreign haplotypes in CSSL‐2626 have changed the start and stop codons of *GH_D03G0443* at the same time, resulting in the simultaneous loss of core transcript components. Meanwhile, the meteoric increase of *GH_D12G2520* (UDPG 6‐dehydrogenase) in CSSL‐2665 (from 0.3 NC in TM‐1 to 266.27 NC) and silencing of *GH_D13G0202* (Actin‐3) in CSSL‐2633 (from 454.79 NC in TM‐1 to 1.09 NC) illustrate the impact of introgression in another respect: In these instances, the foreign haplotype may have activated or promoted an enhancer and silencer, respectively (Figure 3e). The host CSSLs of the three genes demonstrated here significantly differ from the TM‐1 in fibre quality, which empirically support the impact of these genes on fibre quality changes (Figure S11). In total, 182 genes showed dramatic increase and 129 genes showed silencing in expression (Table S10). Ultimately, the substantial genetic variation and extensive transcriptomic changes introduced by foreign segments are what drive fibre differences in the CSSL population.

### Transcriptomic landscape of fibre development in CSSL population

**Figure 4:**
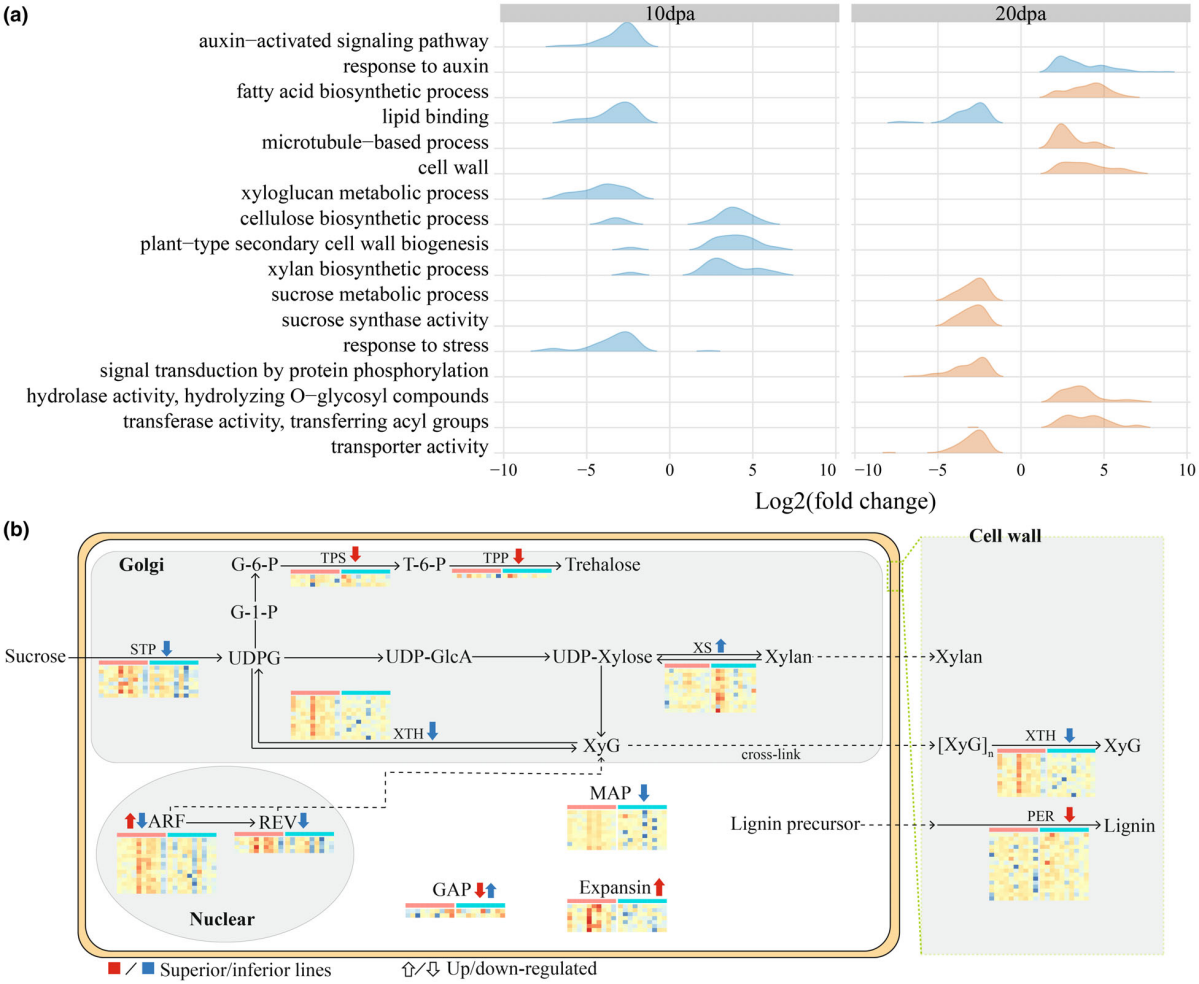
Candidate genes and regulatory pathways involved in fibre development. (a) Gene ontology (GO) enrichment pathways of DEGs in CSSLs with longer (orange) or shorter (blue) FL compared to TM‐1 at 10 DPA and 20 DPA. The *Y*‐axis lists GO terms, the *X*‐axis presents the log fold‐change value indicating the difference of expression between CSSL and TM‐1 for genes enriched in the term and the ridge height indicates the density of genes with the specific expression pattern. (b) Expression patterns of DEGs in key pathways and hotspot genes related to fibre elongation. The direction of the arrow indicates the expression patterns (up‐ or down‐regulated) and the colour indicates the type of line (line with longer or shorter fibre). ARF, Auxin‐response factor; G‐1‐P, Glucose 1‐phosphate; G‐6‐P, Glucose 6‐phosphate; GAP, GTPase‐activating protein; REV, Homeodomain‐leucine zipper (HD‐Zip) proteins class III; TPP, Trehalose‐phosphate phosphatase; TPS, Trehalose‐phosphate synthase; XS, Xylan synthase.

The stable genetic background of CSSL makes it a powerful population for investigating the dynamic changes in fibre transcriptome. For every one of the CSSL that has diverged fibre quality compared to its recurrent parent TM‐1, its transcriptomic changes highlight candidate genes and pathways involved in fibre development. In order to capture the transcriptomic effects of wide‐ranging foreign variation brought by interspecific introgression, particularly the regulatory pathways related to fibre quality, we selected five CSSLs with longer FL and five with shorter FL for comparative transcriptome analysis. In total, 13 855, 6632 and 13 168 DEGs, based on the mapping reads to the TM‐1 reference genome, were identified in fibre at 0 DPA, 10 DPA and 20 DPA. Focusing on the stages of rapid elongation (10 DPA) and secondary cell wall thickening (20 DPA), we found multiple regulatory pathways to exhibit overall differences in expression between superior and inferior CSSLs (Figure 4a). Notably, at 10 DPA, xyloglucan metabolism genes were significantly downregulated in inferior CSSLs. Xyloglucan is the major hemicellulose in the primary cell wall, where it plays a fundamental role in cell wall reorganization and expansion through its ability to bind cellulose, which function requires cellulose synthase and can be influenced by the biological sources of and side chains attached to cellulose and xyloglucan (Chambat *et al*., 2005; Kim *et al*., 2020; Stratilová *et al*., 2020). Typically, genes involved in primary cell wall development are quiescent during the fibre elongation stage in the inferior lines; however, they exhibit highly active expression of genes in plant‐type secondary cell wall biogenesis and related pathways, that is, cellulose and xylan biosynthesis, which synthesize the main hemicellulose of the secondary cell wall. This finding indicates that while the CSSLs with longer FL were still in the rapid elongation stage, the lines with shorter fibre stopped elongation prematurely and entered into thickening the secondary cell wall. Notably, the inferior lines also showed decreased expression of stress response genes, suggesting those lines may be more sensitive to environmental changes. In particular, auxin‐activated signalling was downregulated. A previous study showed that xyloglucan metabolism is induced by auxin signalling in the course of cell expansion (Labavitch and Ray, 1974); combined with the above results, this highlights a regulatory relationship of three biological processes in fibre elongation: auxin signalling, xyloglucan metabolism and cellulose biosynthesis.

As with regulatory pathways revealed by CSSLs with divergent FL, CSSLs with divergent FS and FM also have differential expression genes enriched in auxin signalling, cell wall constitution biosynthesis, sucrose synthesis and transport, and resistance (Figure S12). Except for the auxin‐activated signalling genes, fibres with weaker FS revealed active expression in some genes response to auxin, with most such genes encoding auxin response factor (ARF) proteins and small auxin‐up RNA (SAUR)‐like proteins, respectively. SAUR proteins exhibit specific functions and molecular mechanisms in different stages of plant development in different tissues. In most cases, they act to promote cell proliferation and expansion (Ren and Gray, 2015); however, molecular studies in *Arabidopsis* and rice have also reported negative effects of SAUR overexpression on the synthesis of auxin and auxin transporters (Kant *et al*., 2009; Markakis *et al*., 2013). In this study, expression of genes encoding SAUR‐like and ARF proteins are closely and inversely related in weaker fibre lines; furthermore, the silencing of ARF proteins may also affect cross‐talk between auxin and ethylene, resulting in retardation of fibre development. The lines with coarse fibre (worse FM) again showed higher expression of genes involved in secondary cell wall biogenesis at 10 DPA; interestingly, they also exhibited inactivation of genes involved in xylem development, which regulate the lateral development and polarity of the secondary cell wall (Prigge *et al*., 2005; Zhong and Ye, 2004). Overall, the coarse fibre lines typically exhibited uncoordinated expression of functionally synergistic pathways, and coarse fibres are usually considered to have a shorter development time; these findings may explain why lines with coarse fibre perform poorly in terms of FS and FM, even though they exhibit more active expression of genes related to secondary cell wall development at 10 DPA.

Examining the major DEGs associated with these processes, we found the lines with shorter fibre to also have lower expression of sucrose transport protein (STP) at 10 DPA (Figure 4b), while the lines with longer fibre had relatively lower expression of peroxisome (PER), which implies less reactive oxygen species (ROS) toxicity and less lignin synthesis/polymerization, the latter of which is typically considered to make the cell wall too stiff for fibre cell elongation (Shigeto and Tsutsumi, 2016). The active expression of microtubule‐associated protein (MAP) and genes relating to cell wall structure, such as xyloglucan endotransglucosylase/hydrolase (XTH), in superior fibre lines suggest their elongation advantage may persist longer period compared to the inferior lines. However, we found the genes regarding sucrose transport and utilization to decrease in superior lines at 20 DPA. This phenomenon may be related to fibre developmental stage: superior fibre at that timepoint is in the mid‐late stage of fibre elongation and the initial stage of fibre thickening, while inferior fibre is firmly engaged in secondary cell wall thickening. As a final observation, the relevant genes in protein phosphorylation signal transduction pathway, which are reportedly involved in inducing ATPase phosphorylation and thereby promoting cell elongation (Lin *et al*., 2021), were found to have lower expression in superior fibre at 20 DPA.

### *Cis*‐, *trans*‐expression and regulatory networks of fibre quality improvement in CSSL

**Figure 5:**
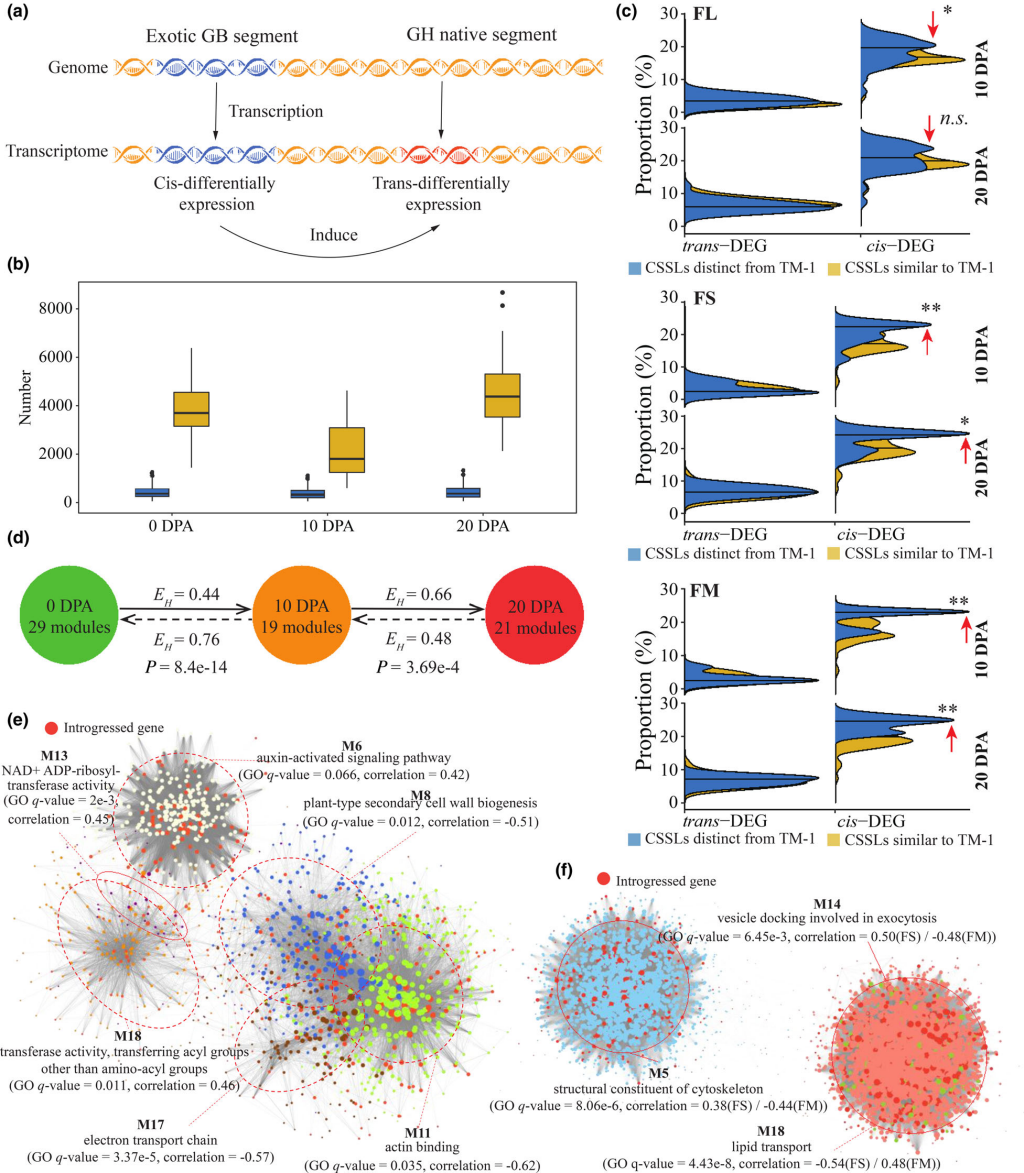
The pattern of *cis‐* and *trans‐*expression and their association with fibre qualities in CSSLs. (a) Illustration of *cis‐*expression and *trans‐*expression. (b) The number of DEGs in the substitution regions (*cis*‐DEGs) and adaptive regions (*trans*‐DEGs) in the CSSL population across 0 DPA ovule, 10 DPA and 20 DPA fibre. (c) Proportion of *cis*‐DEG and *trans*‐DEG in 10 DPA and 20 DPA fibres. A greater proportion of *cis*‐DEGs is clearly observed for CSSLs with significant phenotypic deviations. Mann–Whitney *U*‐test was applied for statistical test, with *P ≤ 0.05, **P ≤ 0.01 and *n.s*. means *P* > 0.05. (d) The number of co‐expressed modules detected in three timepoints and equitability of genes in different networks. Student‐*t* test was applied for *E*_*H*_ differences test. Co‐expression regulatory networks of (e) 10 DPA fibre and (f) 20 DPA fibre, specifically modules with absolute expression correlation higher than 0.4. The primary regulatory pathway of each module and its express correlation with the phenotype were labelled.

Importantly, differential expression of core genes within introgressed segments (*cis*‐DEGs) could alter expression of genes throughout associated pathways, even if those genes are not in the introgression region (*trans*‐DEGs). This effect forms the transcriptional foundation of fibre quality differences in the CSSL population and explains the widespread distribution of DEGs in CSSLs (Figure 5a). Generally, the CSSLs exhibited a wide range of *cis*‐DEG counts: from 3 to 1260 at 0 DPA, 2 to 1119 at 10 DPA and 4 to 1336 at 20 DPA, as revealed by the expression level derived based on CSSL pseudo‐genome. Likewise, the number of *trans*‐DEGs ranged from 1442 to 6383 at 0 DPA, 598 to 4627 at 10 DPA and 2129 to 8676 at 20 DPA (Figure 5b, Table S11). Interestingly, we found CSSLs in which fibre qualities differed significantly from TM‐1 to have higher proportions of *cis*‐DEGs (the number of *cis*‐DEGs divided by all genes introgressed), while the proportion of *trans*‐DEGs was comparable across the population (Figure 5c). This suggests that although all *cis*‐ and *trans*‐DEGs can potentially contribute, differential expression of genes within introgression segments plays a dominant role in the determination of fibre quality (Table S12).

The occurrence of *cis*‐ and *trans*‐DEGs in CSSLs hints at regulatory relationships during fibre development. To further understand the interrelationships of *cis*‐DEGs, *trans*‐DEGs and the divergent regulatory pathways described in the previous section and thereby study their effects on fibre quality, we constructed weighted co‐expression regulatory networks using 20 CSSLs with superior or inferior fibre quality and all 23 384 DEGs across the three developmental stages (Figure S13), this strategy makes the networks more biologically robust and accurate than the ones including all 99 CSSLs (Figure S14). In total, networks constructed from 0 DPA ovule, 10 DPA and 20 DPA fibres yielded 29, 19 and 21 co‐expression modules, respectively. Despite having the largest number of co‐expression modules, the 0 DPA network had only two modules that significantly correlated with fibre quality, in contrast to seven in the 10 DPA and four in the 20 DPA networks, respectively (Figure S15). Further analysis explored the gene flow pattern among modules across the three networks revealed limited conservations (Figure S16), which implies differences in major physiological processes and the genes involved: ovule development and initiation of fibre at 0 DPA, rapid elongation corresponding to primary cell wall development in fibre at 10 DPA and secondary cell wall thickening at 20 DPA, respectively. Moreover, we found that the gene clusters with different co‐expression patterns in 0 DPA usually flow into one or only a few co‐expression modules in 10 DPA (Figures 5d, S17 and S18), with a significantly lower Shannon's equitability index (*E*_*H*_), represents higher concentration levels (Sheldon, 1969), compared to the genes flow from 10 DPA network to 0 DPA network; conversely, the situations are just the opposite for the reciprocal gene flows between 10 DPA and 20 DPA networks. This finding indicates high level of functional specificity in fibre at 10 DPA, while more multifaceted and diverse physiological processes are involved in the 0 DPA ovule as well as 20 DPA fibres.

Based on the significant modules identified in three regulatory networks, we further screened 363 key genes that simultaneously have strong membership (≥0.8) with modules of interests and strong correlations (≥0.5) with fibre qualities (Table S13). Among these genes, 45 genes overlapped with QTL candidate intervals (1 Mb flanking region of significant GWAS signals, Table S13). Although these key genes that co‐localized with QTL exhibited modular enrichment in the specific network, such as 17 genes were found in the M5 of 20 DPA network, and 10 genes were found in the M11 of 10 DPA network, most of these genes have unique shifting patterns across three networks, indicating their specific roles in different developmental stages. Regarding specific gene shifts among the networks (Tables S14 and S15), most genes (11 378 out of 17 024, 66.84%) flow between modules of little correlations with the fibre quality, which considered implicated in upstream and basic regulatory pathways affecting fibre quality formation (Table S16). Nevertheless, many genes involved in the important regulatory pathways aforementioned also reveal the dynamic changes patterns worth noting, such as pattern M15‐M1‐M5 revealed by the genes involved in microtubule‐related pathways, flow from M15 in 0 DPA network to M1 in 10 DPA network and subsequently to M5 in 20 DPA (Table S16). These results illustrate the potential connections between the networks of different stages and highlight the stage‐specific expression patterns in fibre development.

Then, we focused on the key regulatory pathways identified in the 10 DPA and 20 DPA networks as these two stages are more relevant to fibre quality formation, whereas the 0 DPA data regarding the ovule development and fibre initiation are typically considered more relevant to lint yield. In the network constructed from 10 DPA fibre transcriptome data, 19 co‐expressed modules were detected, of which seven modules (M4, M6, M8, M11, M13, M17 and M18) had statistically significant relation to fibre quality (Figure S15). Introgressed genes were widely distributed across those seven modules (red dots, Figure 5e,f), which indicates the fundamental and universal role of introgressed genes in the regulatory network of fibre improvement. The presence of both *cis*‐ and *trans*‐DEGs in these modules also suggests their synergistic regulatory relationship; overall, an average, 73.86% of *cis*‐ or *trans*‐DEGs were present in co‐expression networks, with 4.82% being included in the seven key modules (Figure S19).

We further divided the key modules into two clusters based on their topological relationships and correlation with fibre quality: one cluster comprising modules M6, M13 and M18, which have positive correlations with fibre quality, and the other modules M8, M11 and M17, which have negative correlations with fibre quality (Figure 5e). GO term enrichment analysis indicated genes in the positive cluster to be enriched in transferase activity, NAD+ ADP‐ribosyltransferase activity and auxin‐activated signalling, while genes in the negative cluster were enriched in the terms actin binding, plant‐type secondary cell wall biogenesis and electron transport chain. In the previous section, we found that early thickening of the secondary cell wall may adversely affect the formation of high‐quality fibre; this conclusion is consistent with the negative correlation between expression of genes involved in plant‐type secondary cell wall biogenesis (M8) and fibre qualities observed in this analysis. For genes in M11, the main enriched term was kinase interacting (KIP1‐like) family protein, which are reportedly involved in the initiation of fibre secondary cell wall thickening (Li *et al*., 2020). In general, transcriptome analysis at 10 DPA highlighted the negative impact of earlier initiation of the secondary cell wall on fibre quality, and further indicated physiological responses involving kinases and phosphatases (M17) to interact with this process.

In the regulation network constructed from 20 DPA transcriptional data, we identified 21 co‐expressed modules, among which four were significantly associated with FS and FM (Figures 5f and S15). As in the 10 DPA analysis, most *cis*‐ and *trans*‐DEGs (82.78%) were included in the regulatory network, with 16.01% being in the four FS‐associated modules. Those key modules mainly related to the metabolic process terms structural constituent of cytoskeleton (M5) and vesicle docking involved in exocytosis (M14), which positively correlated with fibre quality, and the terms sucrose and lipid metabolic process (M18 and M3), which negatively correlated with fibre quality. Exocytosis is fundamental to intracellular trafficking, and is connected to several proteins important in fibre development such as microtubules, actin‐binding proteins and scaffolding factors (Li *et al*., 2018). This indicates that vesicle docking and cytoskeletal development are coordinated, and this coordination plays a positive role in fibre quality improvement.

### Kernel regulatory network reveals candidate genes for improving fibre quality

**Figure 6:**
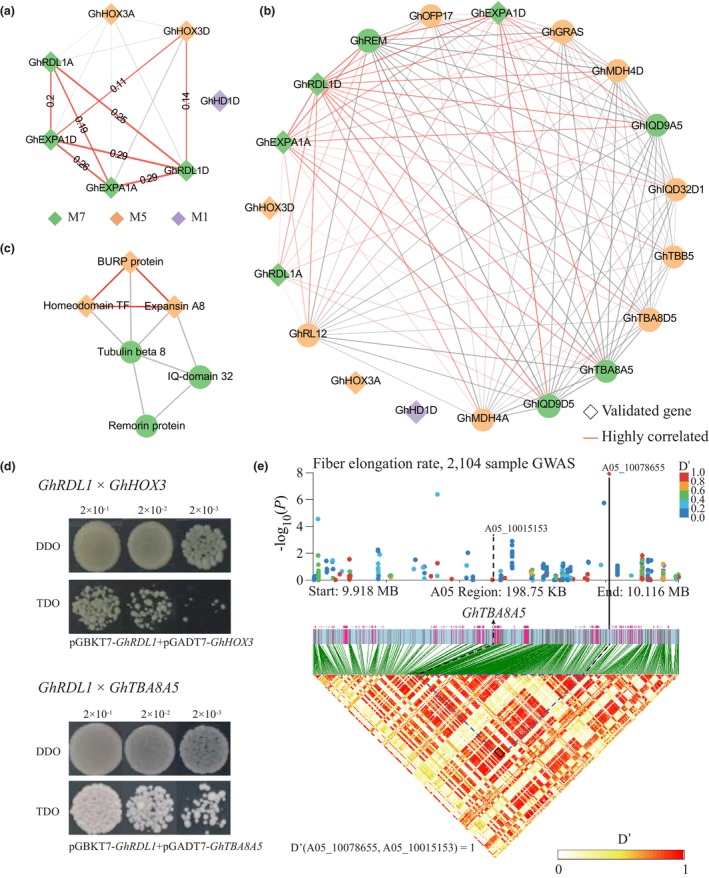
Molecular interactions revealed by the regulatory network. (a) Subnetwork composed of validated genes extracted from the expression network. Interactions with weighted correlation exceeding 0.11 (99th percentile of the whole network) are marked in red line, with their weighted correlations labelled above. Gene symbol colour represents the co‐expression module to which it belongs. (b) Subnetworks of the 12 genes that show high correlations with validated genes. Interactions with weighted correlation exceeding 0.15 and with at least one gene in the gene pairs is a validated gene are marker in red line. (c) Functional annotation for the validated and predicted genes. Interaction lines represent the weighted correlation for the gene‐pair as indicated in the subnetwork. Interactions that have been validated in previous studies are red. (d) Y2H assays testing the molecular interaction of GhRDL1 with GhHOX3 and GhTBA8A5. (e) GWAS analysis of fibre elongation rate in a natural cotton population (upper panel) and LD heat map (lower panel) of the region surrounding *GhTBA8A5*. Pink rectangles in the middle panel represent gene coding regions, and grey lines represent variants.

The regulatory networks constructed in this study also revealed key genes and molecular interactions. Particularly, we validated the network involvement of GhHOX3, a well‐studied homeodomain‐leucine zipper (HD‐ZIP) transcription factor that controls cotton fibre elongation (Shan *et al*., 2014). According to previous studies, GhHOX3 activates RD22‐like1/BURP (GhRDL1), which regulates trichome development and interacts with α‐expansin (GhEXPA1), which in turn functions in cell wall loosening and expansion (Wang *et al*., 2004). GhRDL1 has also been reported to interact with GhEXPA1 in that process (Xu *et al*., 2013). In the regulatory network for 20 DPA fibre, we found strong weighted correlations among the genes encoding these proteins: 0.29 between *GhRDL1D* (*GhRDL1* homologue in D subgenome, similarly hereafter) and *GhEXPA1D*, 0.11 between *GhHOX3D* and *GhEXPA1D* and 0.14 between *GhHOX3D* and *GhRDL1D*. All these values exceed the network‐wide 99th percentile of pairwise correlations (0.11) (Figure 6a, Table S17), indicating the reliability of regulatory network. Another gene encoding a HD‐ZIP IV family protein, *GhHD1*, was included in the subnetwork constructed from *GhHOX3*, *GhRDL1* and *GhEXPA1* to serve as a ‘negative control’. GhHD1 has been demonstrated to affect trichomes, but exerts only a minor effect on fibre development (Walford *et al*., 2012). Appropriately, *GhHD1* showed very little correlation with other key genes in the subnetwork.

To discover genes that contribute to fibre quality improvement, we screened the entire network for genes that correlated highly with the above validated regulators; this yielded an additional 12 genes (Figures 6b, and S20). Three of the 12 candidate gene encode tubulin, which has long been recognized as involved in fibre elongation and strengthening (Janke and Magiera, 2020) (Figure 6c, Table S18); another three encode IQ‐domain protein, a calmodulin‐like protein that stimulate actin cytoskeleton changes mediated by proteins such as myosin, and a recent study in rice also reported its function in affecting the rearrangement of cortical microtubules (Yang *et al*., 2020). Another candidate gene featured a gibberellic‐acid insensitive, repressor of GAI, and scarecrow (GARS) domain that could form complexes with calcium and calmodulin‐dependent kinase to induce downstream genes and regulate diverse development processes, such as root development and hormone signalling (MacLean *et al*., 2017). Additionally, the candidate genes included a remorin family protein that may function in virus defence and plasmodesmata biogenesis (Raffaele *et al*., 2009; Zhuang *et al*., 2020). Silencing of *GhHOX3* altered the expression of 10 candidate genes, with each displaying either a significant decrease or slight increase in expression (based on 6 DPA fibre RNA‐Seq of a *GhHOX3*‐silenced line, provided in Shan *et al*., 2014). This observation further validates the reliability of the constructed regulatory networks and suggests the potential of these genes in enhancing fibre qualities (Table S18). In the CSSLs, the candidate genes exhibit expression patterns similar to those of the validated genes, and hence exhibit high correlation with fibre quality traits, especially for the tubulins *GhTBA8D5* and *GhTBA8A5* (Figure S21). We additionally validated and confirmed the molecular interaction of GhTBA8A5 and GhRDL1 by yeast two‐hybrid assay (Figure 6d). And we also screened a natural population for variation in *GhTBA8A5* that is significantly associated with fibre quality. This revealed *GhTBA8A5* to be located within the linkage disequilibrium (LD) block tagged by variant A05:10078655, the genotype of which is significantly associated with fibre elongation rate (public data set, Figure 6e).

## Discussion

### CSSLs are excellent resources for unravelling complex traits

Genetic dissection of complex quantitative traits in crops usually requires establishing a specific population to capture the segregation of QTLs. Distinct from conventional mapping populations such as second filial generation (F_2_) and recombinant inbred lines (RIL), near‐isogenic CSSLs feature genetic backgrounds that are identical to the recurrent line except for a few genomic segments, and hence phenotype performance can be straightforwardly attributed to the segregation of one or a few QTLs. This clear inheritance makes CSSLs ideal material in genetic, molecular and breeding studies. Early research involving CSSLs typically employed only one or a few lines for fine mapping in QTL and gene discovery studies. In recent years, with the advancement of multi‐omics and sequencing technologies, an increasing number of crop CSSL populations have been cultivated, and large‐scale transcriptomic and metabolomic studies based on such populations are gradually emerging (Coombes *et al*., 2023; Ecke *et al*., 2015; Guo *et al*., 2013; Li *et al*., 2019; Lopez‐Zuniga *et al*., 2019; Schauer *et al*., 2006; Wang *et al*., 2012a, 2012b; Xi *et al*., 2006). In this study, GWAS found several potential QTLs that related to fibre qualities (FL, FS and FM), of which 17 QTLs are located in genomic regions identified in previous studies, and 14 QTLs are newly found in this CSSL population. The genetic contributions of these novel QTLs are significantly higher than those of the previously reported QTLs, which implies the process of breeding these CSSLs has reintroduced important variations lost in *G. hirsutum*. For crops with narrow genetic diversity, the construction and evaluation of CSSLs is a reliable approach for optimizing agronomic traits. Through transcriptomic analysis of this CSSL population, we identified comprehensive candidate pathways involved in fibre quality development and validated the molecular interactions revealed by the kernel subnetwork. These results highlight CSSLs as a powerful genetic panel for dissection of complex traits.

### Accurate detection of substitution segments in CSSLs

Precise localization of the foreign substitution segments in CSSLs is fundamental for subsequent analysis; yet methods for detecting these artificially introgressed segments are only infrequently described. Common introgression detection methods, such as rIBD and ABBA‐BABA methods (Dasmahapatra *et al*., 2012; Wang *et al*., 2019b), which detect natural introgression between populations or species based on allele frequency or genetic distance, are not applicable for detecting the artificial introgression in CSSLs. Recent CSSL study which transferred *Ambylopyrum muticum* segments into wheat, detected the introgression based on the fact that, due to sequence divergence, the donor segments are different to be aligned with the wheat genome and therefore have off‐cliff coverage dropping (Coombes *et al*., 2023). Coverage‐based method works well for CSSL constructed from species with rather far genetic distant; yet, it is less sensitive for CSSLs constructed from closely related species such as the cotton CSSLs in this study (Figure S22). In light of the clear pedigree that CSSLs possess, we constructed a systematic method based on the rules governing allele transmission across generations and proposed an effective software SSD for accurate identification of substitution segments. The results of our analysis demonstrate that this method can accurately detect foreign substitution segments in CSSLs, even within natural introgression regions, that is, regions that are highly homologous between parental *G. hirsutum* and *G. barbadense* lines. Here, we further explicate the reliability of our methodology from both theoretical and molecular experimental perspectives. In theory, the expected proportion of substitution segments can be calculated based on the number of backcross generations, that is, 50% fewer with every backcross. In our study, 52 of the 99 CSSLs were derived from 3 to 79, with most of these lines obtained through selfing of BC_4_, while the other 47 CSSLs were derived from Hai7124, mostly via selfing from BC_5_. Therefore, the theoretical expected substitution proportion is 0.023 (52/99*0.5^5^ + 47/99*0.5^6^). Across the CSSL population, the 735 substitution segments amount to a total length of 6620.38 Mb. Consequently, the observed average substitution proportion is calculated as 0.029 (6620.38/99/2295.26, with 2295.26 [Mb] being the genome size of TM‐1). In practice, pre‐experiments using InDel and SSR markers have revealed substitution segments covering 72.7% of *G. barbadense* acc. 3–79 (Cheng, 2017), which is also very close to the estimated proportion of foreign introgressed genes in the 52 3–79‐introgressed CSSLs, 72.23% (Figure 3a). The consistency between these theoretical and observed substitution proportions corroborates the success of this population design and the accuracy of substitution detection. Our method is therefore a useful reference for subsequent studies in crop CSSLs.

### Expression patterns of CSSLs

Large‐scale transcriptomic profiling of CSSL fibres in our study indicated the introgressed foreign genes to have generally high expression capacity. Especially, those introgressed genes having orthologues in the TM‐1 genome exhibited expression capacity very similar to native TM‐1 genes. Expression incompatibility was primarily observed in new‐to‐TM‐1 genes, but even those demonstrated an acceptable expression capacity (50.27% on average) in their new genetic environment. Previous studies have shown genes from distant species to exhibit notable expression incompatibility, resulting in limited expression of foreign genes after their introduction (Coombes *et al*., 2023). This phenomenon is likely attributable to the significant genetic disparity typical between parental species, which presents challenges in achieving functional compatibility at the molecular level and therefore, to a certain extent, hinders the development and utilization of CSSLs. Consequently, the selection of parents for CSSL construction should strike a balance between diversity reintroduction and genomic/transcriptomic affinity. Ultimately, the combination of genome‐level introgression with high expression capacity of the introgressed genes makes it possible to study the downstream functions of almost all desirable genomic segments from the CSSL population in question as well as secondary populations further developed.

Another interesting observation is that no definitive correlation of phenotype in the CSSL population with the length of the introgressed segment, the number of foreign introgressed genes or even gene expression capacity (Table S12). For the length of introgression segments and the number of introgressed genes, the establishment of significant phenotypic correlation depends on whether longer segments or more genes encompass more QTLs or key genes, which is obviously variable. As for expression capacity, although we observed significant variation in homologue and novel genes, no clear relationship with phenotypes was demonstrated. This outcome concurs with the conception that heterologous protein expression is principally governed by internal features, that is, mRNA and protein structures, and by codon usage bias specific to the species (Ullrich *et al*., 2015). When comparing plants across identical cultivation environments, the expression capacity of minor but pivotal genes may exhibit strong correlation with phenotypic variation; nonetheless, the general expression capability of genes within all introgressed segments is predominantly influenced by internal and conserved factors. Instead, we discovered a positive correlation between the proportion of *cis*‐DEGs and fibre quality. This finding highlights the fundamental role of *cis*‐regulation in CSSLs and provides new insights into the transcriptomic basis of the phenotypic divergence observed between CSSLs and parent lines.

### CSSL population with whole‐genome level of introgression helps cotton genetic breeding and molecular regulation research

Cotton stands as the most important source of natural fibres and has a wide range of demonstrated applications. Modern cotton cultivars mainly belong to *G. hirsutum* and *G. barbadense*. *G. hirsutum* has high lint yield with medium fibre quality, while *G. barbadense* is just the opposite with superior fibre quality but moderate lint yield. Despite the high homology between *G. hirsutum* and *G. barbadense*, genome studies in recent years have revealed considerable structural variations and numerous short sequence polymorphisms between the species, which variations are considered to be the main source of their divergence in field features. Over the centuries, cotton has undergone strong artificial selection, especially for *G. hirsutum* cultivars, which continuous selection has led to a strong genetic bottleneck. In short, the lint yield of *G. hirsutum* cultivars is increasing, but their genetic diversity is gradually being lost. Through gene recombination and chromosomal crossover, we are able to introduce genomic segments from *G. barbadense* into the *G. hirsutum* genome, which not only breaks up the strong LD, but also reintroduces quality‐related genes into *G. hirsutum*. In this study, we selected 99 CSSLs developed on the *G. hirsutum* background and incorporating *G. barbadense* genomic segments that collectively represented the majority (87.25%) of the donor genome. Leveraging the high‐quality reference genomes available for the parental cotton varieties TM‐1, 3–79 and Hai7124, we then constructed a unique ‘pseudo‐genome’ for each CSSL. This enabled us to accurately identify the foreign substitution sequences and precisely quantify each transcriptome based on the exogenous gene sequences. The 99 CSSLs constructed in this study thus provide valuable genetic resources for multi‐trait polymerization breeding and the molecular study of cotton.

Recently, a large number of transcriptome studies have provided inspiration for the improvement of cotton fibre. In particular, comparative transcriptomic analysis between several short‐fibre or fibreless cotton lines and wild cotton has revealed many key genes and transcription factors involved in fibre development, such as *MYB*, *bHLH* and *HD‐bZIP*, and regulatory pathways such as hormone signalling, cellular energy metabolism and fatty acid metabolism (Jan *et al*., 2022). This study focused on fibre elongation and secondary cell wall thickening stages, for which some of the findings were similar: differential expression between superior and inferior CSSLs of regulatory pathways related to carbohydrate metabolism, hormone signal transduction and fatty acid metabolism, and upregulation in superior lines of many key genes such as tubulin and expansin, which are involved in loosening and expansion of the cell wall. Interestingly, inferior CSSLs exhibited high expression of cellulose and hemicellulose synthesis genes involved in the process of secondary cell wall thickening during the rapid elongation stage, which implied early termination of fibre elongation to have a negative impact on fibre quality. This result was confirmed by network analysis: The M8 module with genes enriched in plant‐type secondary cell wall biogenesis has a negative correlation of −0.51 with fibre quality, and the KIP1‐like family protein in M11 is suggested to be closely related to the initiation of this process. Moreover, we found superior lines and inferior lines to differ in the synthesis and transport of sucrose substances and the phosphorylation process. Further network analysis indicated that phosphorylation most likely affects fibre quality through phosphokinases regulating the initiation of secondary cell wall thickening, based on KIP1‐like proteins being included in the M11 module and that module's close topological relationship with M8. The network analysis in this study simplified the complex transcriptional relationships between genes, and the analysis of specific co‐expression modules clarified key pathways and their relationships during fibre development. Further molecular exploration of the interaction network between gibberellic acid, tubulin, calmodulin‐like protein and calmodulin‐dependent kinase, along with that network's role in cell wall elongation and thickening, should be beneficial in further elucidating the physiological mechanisms that underlie the improvement of cotton fibre quality.

## Materials and methods

### Development of cotton CSSL population and their phenotyping

The genetic standard *G. hirsutum* line, TM‐1, was used as the recurrent parent to cross with *G. barbadense* acc. 3–79 and cv. Hai7124, and the resulting F_1_ population was backcrossed with TM‐1 four to five times. Finally, the BC_4:5_ population was self‐pollinated for four generations to develop 166 3–79 CSSLs and 174 Hai7124 CSSLs. Marker‐assisted selection (MAS) was applied to BC_n_ and BC_n_F_n_ to track the substitution segments. Based on the phenotypic data, we selected 52 3–79 CSSLs and 44 Hai7124 CSSLs with lint yield or fibre quality significantly different from TM‐1. More details about cultivation period and location can be obtained in Appendix S1.

The 99 CSSLs and their parental lines were planted at Dangtu from 2018 to 2020 for phenotyping of three important fibre quality measures, FL, FS and FM, with three biological replicates. For phenotypic data across multiple years, the best linear unbiased estimation (BLUE) of the genotypic component was calculated and used as phenotype in the following analysis.

### Detection of substitution segments using genomic variations

Whole‐genome SNP markers were used for the detection of substitution segments in CSSLs. The detection strategy comprised three main steps: allele classification, slide window analysis and ambiguous allele adjustment. We firstly determined whether each loci in the CSSL was lineage‐specific and clarified its parental origin by rules governing allele transmission across generations, then we calculated the proportion of loci originating from the donor parent (local substitution proportion, LSP) in each (sliding) window, and finally, we corrected the regions with high proportion of loci of ambiguous origin if most of the remaining alleles are donor lineage‐specific. A genomic segment is identified as substitution of *G. barbadense* if it has updated substitution proportion exceeding 80%. More details are described in Appendix S1.

### Two reference genomes utilized during the transcriptome quantification

First, the TM‐1 reference genome was employed directly for gene expression analysis, allowing us to obtain a unified expression panel of the CSSL population for subsequent analyses. The second reference genome was a pseudo‐genome specific to each CSSL, constructed from the TM‐1 genome by replacing the regions corresponding to substitution segments with their highly conserved counterpart sequences from *G. barbadense* acc. 3–79 or cv. Hai7124 (Figure S9). This strategy enabled us to conduct precise investigations of individual‐level transcriptomes. The technical details for constructing the pseudo‐genome are as follows: (1) the genomic sequences of substitution segments detected by SSD were extracted from TM‐1 genome, and all variants (including SNPs, Indels and structural variations) were substituted to obtain the consensus sequences for each segment. This step is implemented by bcftools with the subcommand *consensus* (Li, 2011); (2) the consensus sequences were blasted against the either *G. barbadense* acc. 3–79 or cv. Hai7124 genome (Hu *et al*., 2019; Wang *et al*., 2019a) to get their ‘original copies’ (Altschul *et al*., 1990); (3) the *G. barbadense* segments that homologous to the consensus sequences were substituted into TM‐1 genome, thereby individual level of CSSL pseudo‐genome is constructed.

### Identification of *cis*‐DEGs and *trans*‐DEGs

In this analysis, *cis*‐genes refers to genes located within the introgressed segments, while *trans*‐genes refer to those located outside of introgressed segments, that is, genes from the TM‐1 genome. *Cis*‐DEGs are those *cis*‐genes differentially expressed in CSSLs compared to TM‐1. There are two scenarios in which a *cis*‐gene can be identified as a *cis*‐DEG. The first scenario is when the gene does not have an orthologue in the substituted segment, meaning it is a novel gene introgressed from *G. barbadense*, and it is expressed in the CSSL (normalized count ≥1). The second scenario is when the gene has an orthologue within the substituted segment, for these genes, as well as *trans*‐genes, the DEGs are assigned if they have absolute log fold‐change in expression relative to its TM‐1 orthologue equal to or greater than 2 with *P* <0.05.

### Measurement of the equitability level of gene flows among the networks

Shannon's equitability index (*E*_*H*_) is commonly used to characterize the distribution equitability of species with given abundance. In this study, we used *E*_*H*_ to measure if genes in a module are evenly assigned to different modules or centralized flow to one or a few modules in another network. *E*_*H*_ ranges from 0, indicating that all genes in a module flow into just one module in another networks, to 1, indicating that all genes in a module are completely evenly distributed in all modules in another network. For genes in module *i* identified in network A, we firstly calculated their proportions in every *j*‐th module of network B,

$$p_{\mathrm{ij}}=n_{\mathrm{ij}}/n_{j}$$

in which $n_{\mathrm{ij}}$ is the number of genes simultaneously detected in module *i* and *j*, and $n_{j}$ is the total number of genes that contained in module *j*. Then, the equitability level of genes in module *i* from network A to B is

$$E_{\mathrm{Hi}}=-\frac{1}{\ln N_{B}}\sum_{j}p_{\mathrm{ij}}\ln p_{\mathrm{ij}}$$

in which $N_{B}$ is the total of modules identified in network B, and $\ln N_{B}$ represents the maximum diversity of module *i*.

### Data analysis

Methods for the routine analyses, including sequencing details, quality control and downstream analysis are described in Appendix S1.

## Author contributions

TZZ conceived and designed the project. ZFS carried out variety cultivation and field management. ZFS, YH, LF and FD inspected the phenotype data, ZFS and YH collected and prepared the sequencing samples. GAQ conducted the data analysis and interpretation. LSX and ZGH performed the experiment. GAQ and TZZ initiated the draft of the manuscript. All the authors discussed the results and commented on the manuscript.

## Competing interest statement

The authors declare no conflict of interests.

## Codes accessibility

The software SSD proposed in this study and its source codes are freely available at Github repository (https://github.com/GuoanQi1996/SSD).

## Supporting information


**Figure S1** Construction and phenotypic evaluation of the CSSL population.
**Figure S2** Method of detecting substitution segments in the CSSL.
**Figure S3** The distribution of substitution segments number (left) and size (right) detected in 99 CSSLs.
**Figure S4** The numbers of substitution segments at 26 chromosomes.
**Figure S5** The lengths of total substitution segments that detected on 26 chromosomes.
**Figure S6** Comparison of FL between CSSLs with different D13:8088384 haplotypes.
**Figure S7** Haplotype analysis for chromosome D03 in the CSSL population.
**Figure S8** Functional annotation of genomic variants in CSSL population.
**Figure S9** The construction pipeline of CSSL pseudo‐genome.
**Figure S10** The relationship between expression capacity of foreign introgressed genes across 99 CSSLs and fibre qualities.
**Figure S11** Phenotypic performance of fibre quality for CSSLs with specific genes influenced by foreign introgressed alleles, corrsponds to Figure 3d.
**Figure S12** Gene ontology (GO) enrichment pathways of DEGs in CSSLs with superior (orange) or inferior (blue) FS (upper panel) and FM (bottom panel) compared to TM‐1 at 10 DPA and 20 DPA.
**Figure S13** Weighted co‐expression regulatory networks constructed for 0 DPA ovlue (up), 10 DPA (middle) and 20 DPA (bottom) fibre.
**Figure S14** The comparison between co‐expressed modules of 10 DPA fibre identified from all 99 CSSLs (up band) and 20 CSSLs (down band) with divergent fiber quality.
**Figure S15** Pearson's correlations between co‐expression modules of 0 DPA (left), 10 DPA (middle) and 20 DPA (right) networks and fibre quality traits.
**Figure S16** Gene flow patterns of modules in networks constructed from 0 DPA ovlue, 10 DPA and 20 DPA fibre.
**Figure S17** The correspondence of genes in the networks contrcuted from 0 DPA and 10 DPA transcriptomic data.
**Figure S18** The correspondence of genes in the networks contrcuted from 10 DPA and 20 DPA transcriptomic data.
**Figure S19** *Cis*‐ and *trans*‐DEGs included in the co‐expression regulatory network.
**Figure S20** Number of genes that simultaneously interacted with validate genes in 20 DPA co‐expression regulatory network.
**Figure S21** Pearson correlation between the expression levels of the 19 genes of interests and fibre qualities.
**Figure S22** Detecing substitution segments in CSSL‐2656 using coverage‐based method as described by Coombes *et al*.

**Table S1** Phenotypic variabilities of 174 Hai7124 CSSLs.
**Table S2** Phenotypic variabilities of 166 3–79 CSSLs.
**Table S3** Phenotype of FL, FS and FM in the CSSL population and their parental lines.
**Table S4** Summary statistics of chromosomal substitution segments in the CSSLs.
**Table S5** The number and summed length of substitution segments in each chromosome.
**Table S6** The 26 independent SNP markers/genomic regions that significantly associated with FL.
**Table S7** The 23 independent SNP markers/genomic regions that significantly associated with FS.
**Table S8** The 23 independent SNP markers/genomic regions that significantly associated with FM.
**Table S9** Expression capacity (the proportion of genes expressed) of genes at three timepoints across 99 CSSLs.
**Table S10** Impact of substitution segments on the CSSL transcriptome.
**Table S11** The number of *cis*‐ and *trans*‐DEG in 0 DPA ovule, 10 DPA and 20 DPA fibre in the CSSL population.
**Table S12** The Pearson correlations between features of CSSL population and fibre quality performance.
**Table S13** The kernal genes identified in the key regulatory modules that significantly related to the fibre qualities.
**Table S14** Genes flow pattern across networks constrcuted from 0 DPA ovlue, 10 DPA and 20 DPA fibre.
**Table S15** The number of genes with specific gene flow pattern.
**Table S16** The enrichment analysis for genes with specific gene flow pattern across the three networks.
**Table S17** The weighted correlations of validated genes in the development of cotton fibre.
**Table S18** Function annotation of the predicted genes and their expression correlations with fibre quality traits.
**Table S19** RNA‐Seq information for all 839 samples.

**Appendix S1** Supporting Methods.

## Data Availability

The Illumina genomic sequencing data for CSSL population are available at NCBI, Sequence Read Archive (SRA; https://www.ncbi.nlm.nih.gov/) with identifier PRJNA1073590.
