# Supplementary material for: Unravelling the genetic basis and regulation networks related to fibre quality improvement using chromosome segment substitution lines in cotton
Source: Plant Biotechnol J. 2024 Jul 24;22(11):3135–50. doi: 10.1111/pbi.14436 (PMC11500987; doi:10.1111/pbi.14436)
Supplement: Supplementary file 1 — Figure S1 Construction and phenotypic evaluation of the CSSL population. Figure S2 Method of detecting substitution segments in the CSSL. Figure S3 The distribution of substitution segments number (left) and size (right) detected in 99 CSSLs. Figure S4 The numbers of substitution segments at 26 chromosomes. Figure S5 The lengths of total substitution segments that detected on 26 chromosomes. Figure S6 Comparison of FL between CSSLs with different D13:8088384 haplotypes. Figure S7 Haplotype analysis for chromosome D03 in the CSSL population. Figure S8 Functional annotation of genomic variants in CSSL population. Figure S9 The construction pipeline of CSSL pseudo‐genome. Figure S10 The relationship between expression capacity of foreign introgressed genes across 99 CSSLs and fibre qualities. Figure S11 Phenotypic performance of fibre quality for CSSLs with specific genes influenced by foreign introgressed alleles, corrsponds to Figure 3d. Figure S12 Gene ontology (GO) enrichment pathways of DEGs in CSSLs with superior (orange) or inferior (blue) FS (upper panel) and FM (bottom panel) compared to TM‐1 at 10 DPA and 20 DPA. Figure S13 Weighted co‐expression regulatory networks constructed for 0 DPA ovlue (up), 10 DPA (middle) and 20 DPA (bottom) fibre. Figure S14 The comparison between co‐expressed modules of 10 DPA fibre identified from all 99 CSSLs (up band) and 20 CSSLs (down band) with divergent fiber quality. Figure S15 Pearson's correlations between co‐expression modules of 0 DPA (left), 10 DPA (middle) and 20 DPA (right) networks and fibre quality traits. Figure S16 Gene flow patterns of modules in networks constructed from 0 DPA ovlue, 10 DPA and 20 DPA fibre. Figure S17 The correspondence of genes in the networks contrcuted from 0 DPA and 10 DPA transcriptomic data. Figure S18 The correspondence of genes in the networks contrcuted from 10 DPA and 20 DPA transcriptomic data. Figure S19 Cis‐ and trans‐DEGs included in the co‐expression regulatory network. Figure [file PBI-22-3135-s002.pdf]

Supporting Figures for

Unraveling the genetic basis and regulation networks related to fibre quality improvement using chromosome segment substitution lines in cotton

Guoan Qi[1,2], Zhanfeng Si[2], Lisha Xuan[2], Zegang Han[2], Yan Hu[1, 2], Lei Fang[1, 2], Fan Dai[2], Tianzhen Zhang[1,2,*]

Correspondence: *Tianzhen Zhang (cotton@zju.edu.cn)

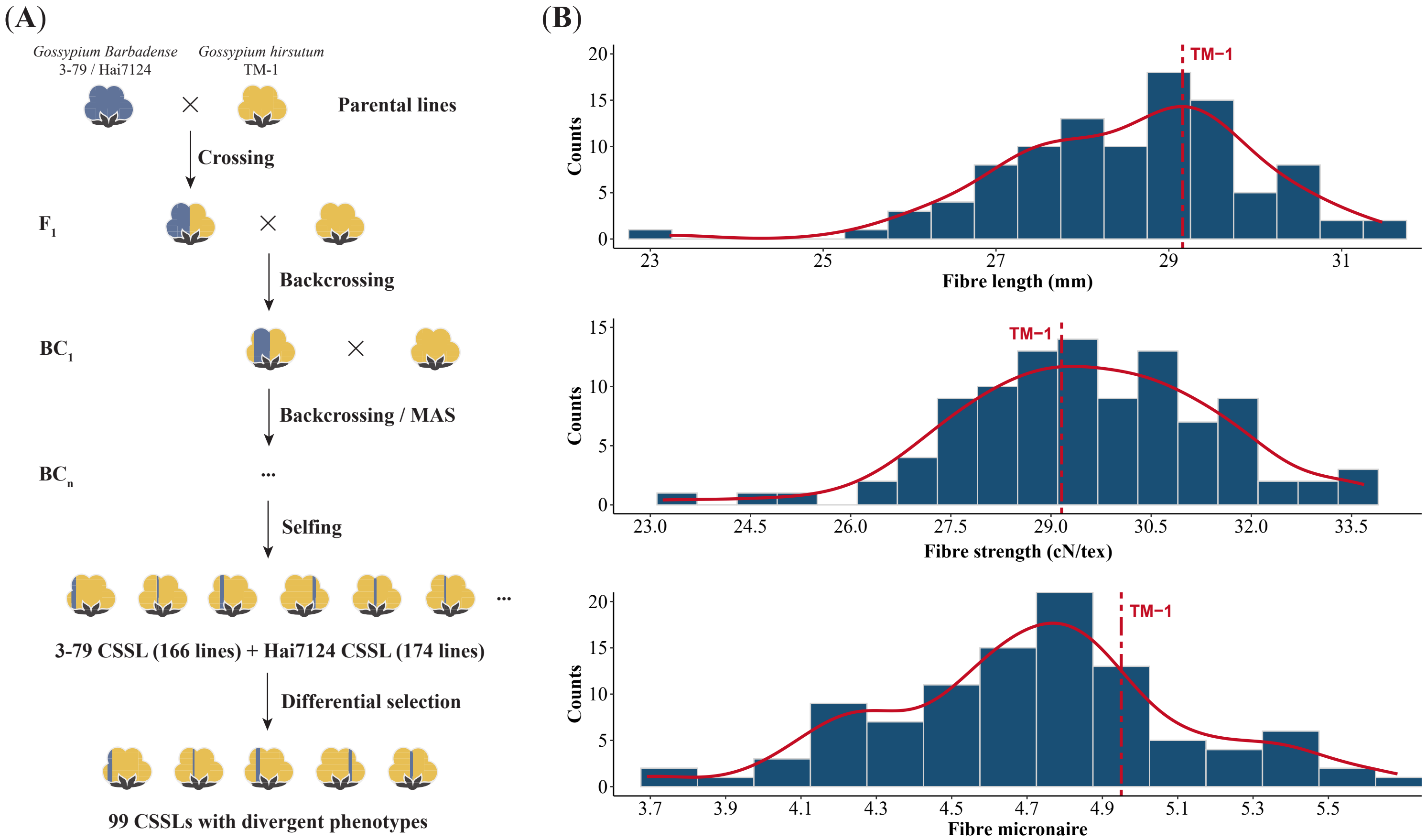

Figure S1. Construction and phenotypic evaluation of the CSSL population. (**A**) Construction of the CSSL population. (**B**) Phenotypic evaluation of fibre length (FL), fibre strength (FS) and fibre micronaire (FM) in the CSSL population.

① Allele classification

Donor line	Recurrent line	CSSL	Class
AA	AA	Aa	RA (Unknown source, redundant)
…	…	…	…
AA	Aa	aa	Recurrent LSA (Gh haplotype)
Aa	AA	aa	Donor LSA (Gb haplotype)
…	…	…	…
Aa	Aa	AA	NLSA (Ambiguous haplotype)
…	…	…	…

② Sliding window analysis

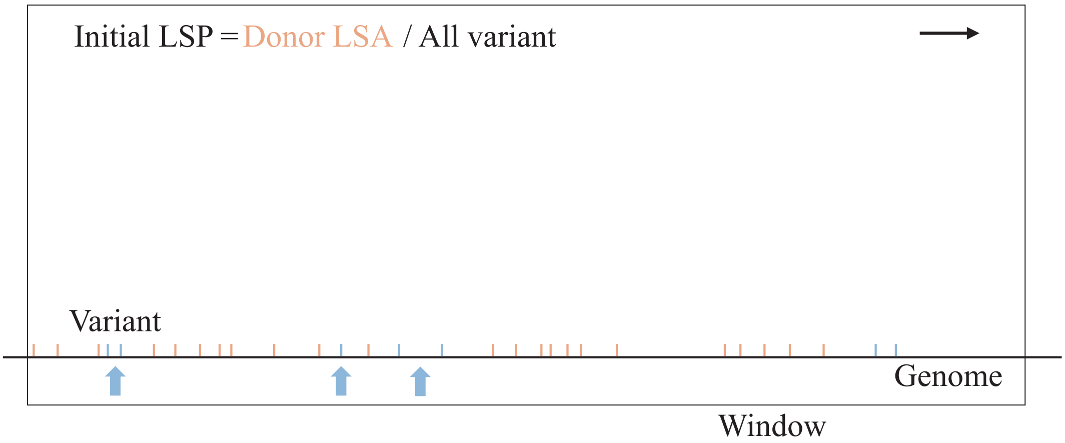

③ Adjustment for NLSA

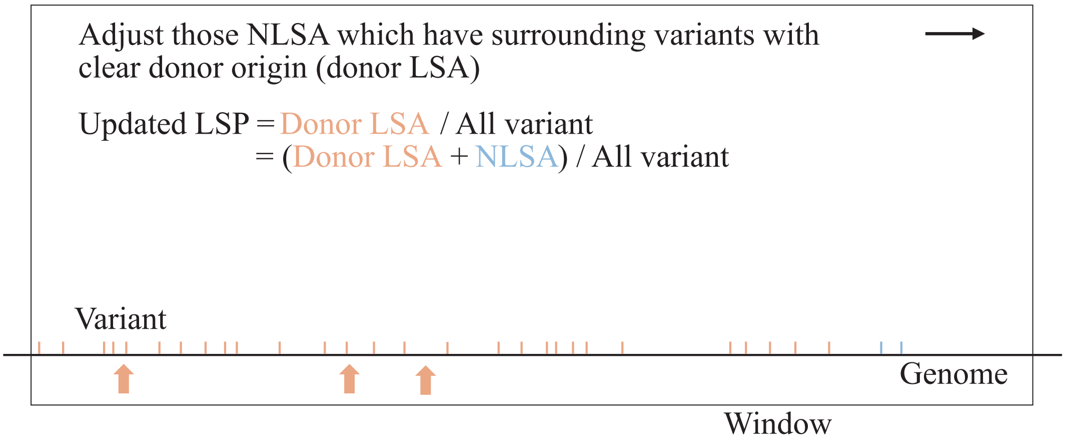

Figure S2. Method of detecting substitution segments in the CSSL. We initially determined the allelic origins of each variation in CSSL according to the laws of allele transmission. Thereafter, we specified the origins of genomic regions via sliding window analysis. In the process, we corrected the origins of the ambiguous haplotype and reassigned the updated substitution proportion for each genomic window.

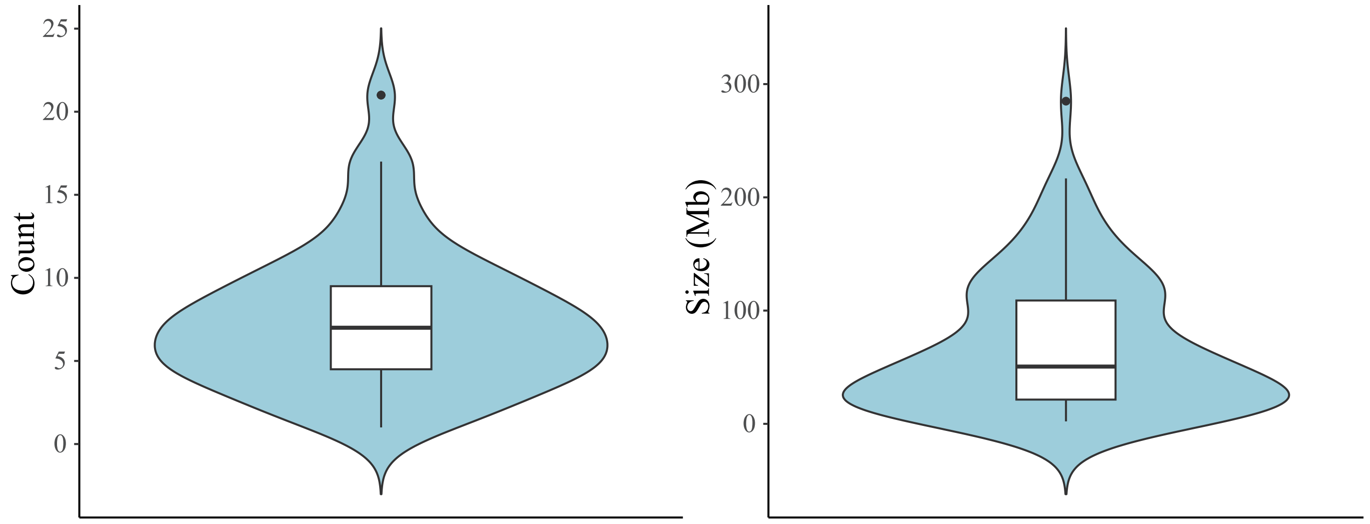

Figure S3. The distribution of substitution segments number (left) and size (right) detected in 99 CSSLs.

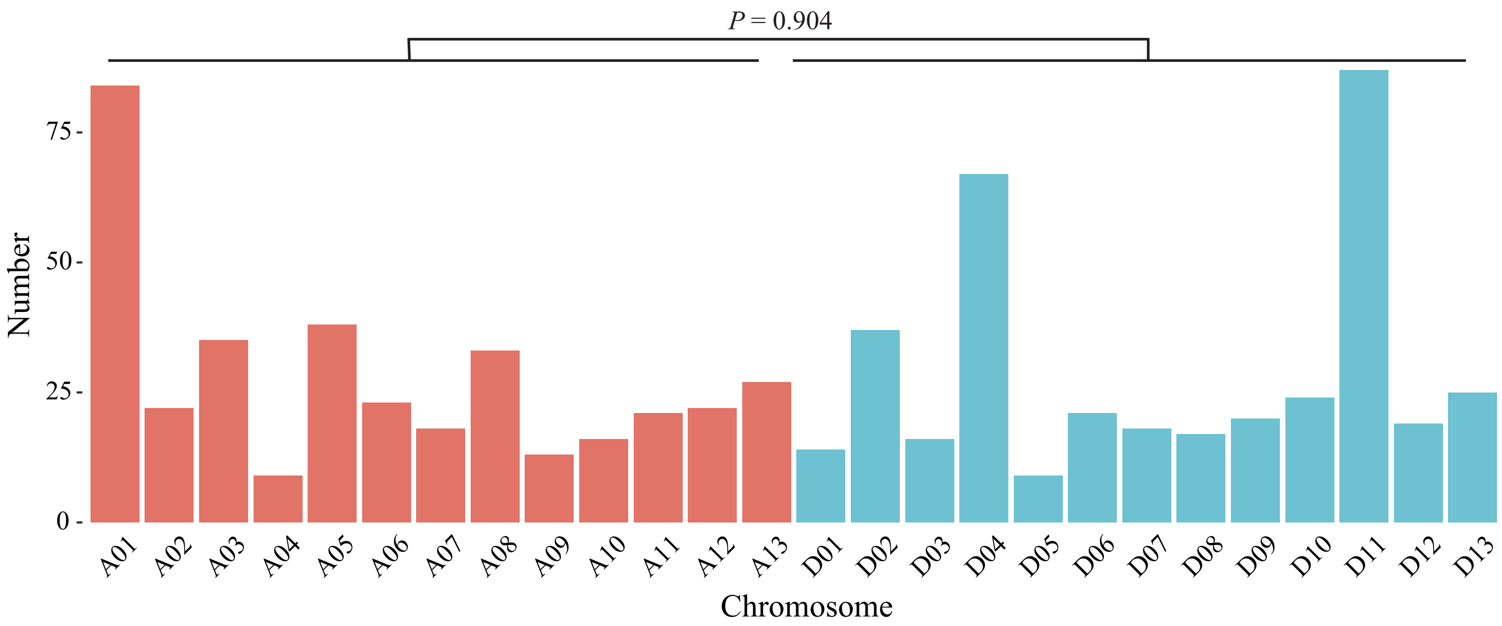

Figure S4. The numbers of substitution segments at 26 chromosomes. The segments numbers at A sub-genome and D sub-genome have no statistical difference. Student-t test was applied for the statistical inference.

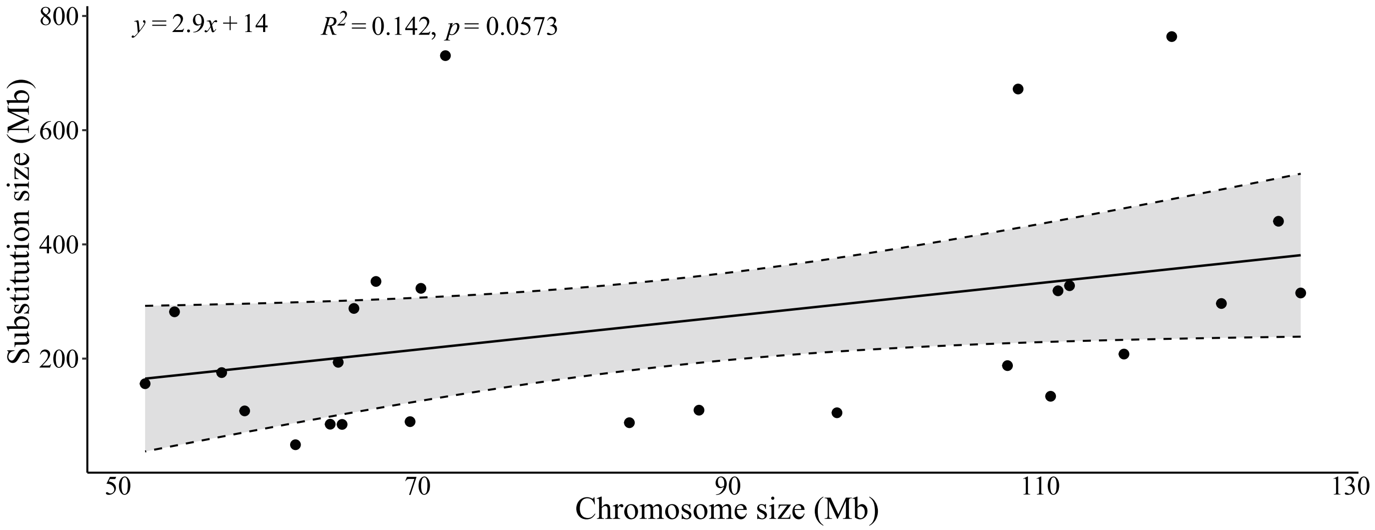

Figure S5. The lengths of total substitution segments that detected on 26 chromosomes. The summed length of the substitution segments on the chromosome is positively correlated with the length of the chromosome itself, with Pearson-correlation 0.38, which indicates that the substitution segments is evenly distributed on the chromosomes. T-test was applied in the linear regression for statistical inference.

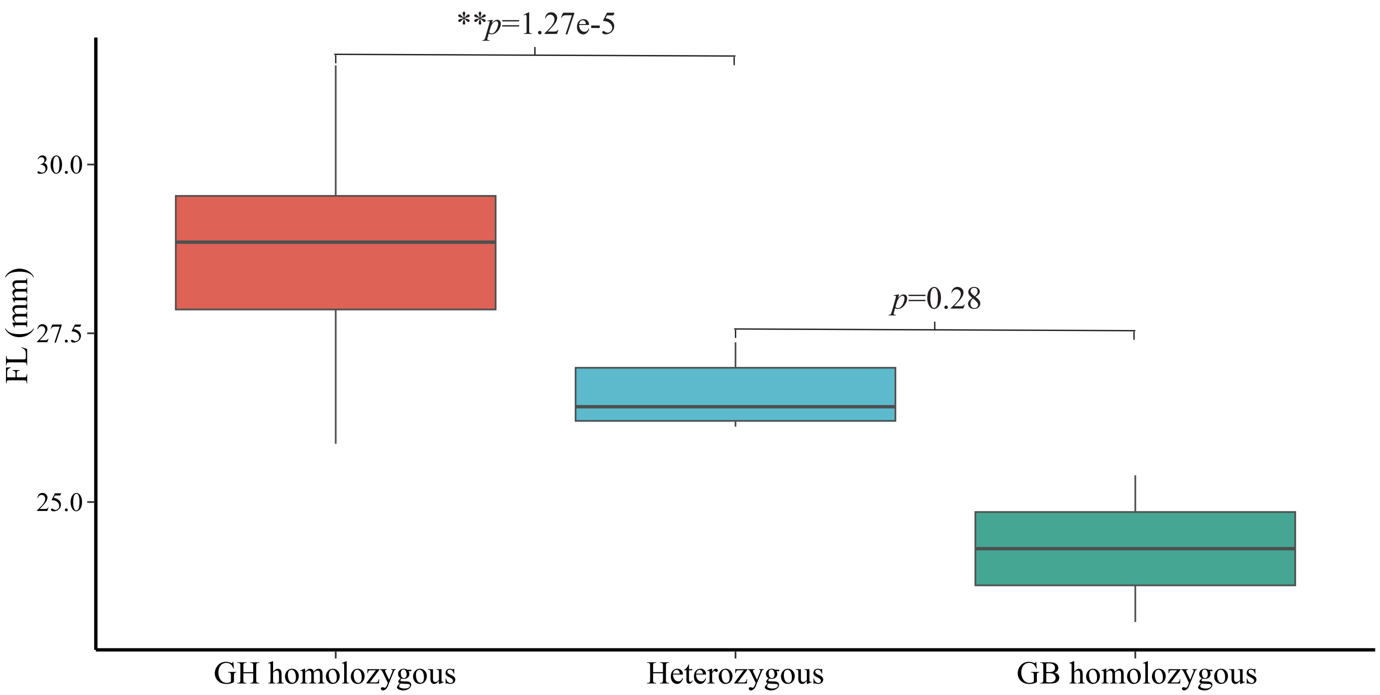

Figure S6. Comparison of FL between CSSLs with different D13:8088384 haplotypes. GWAS analysis shows extremely genetic contribution of GB-haplotype, with each able to reduce FL by 2.11 mm. Student-t test was applied for the statistical inference.

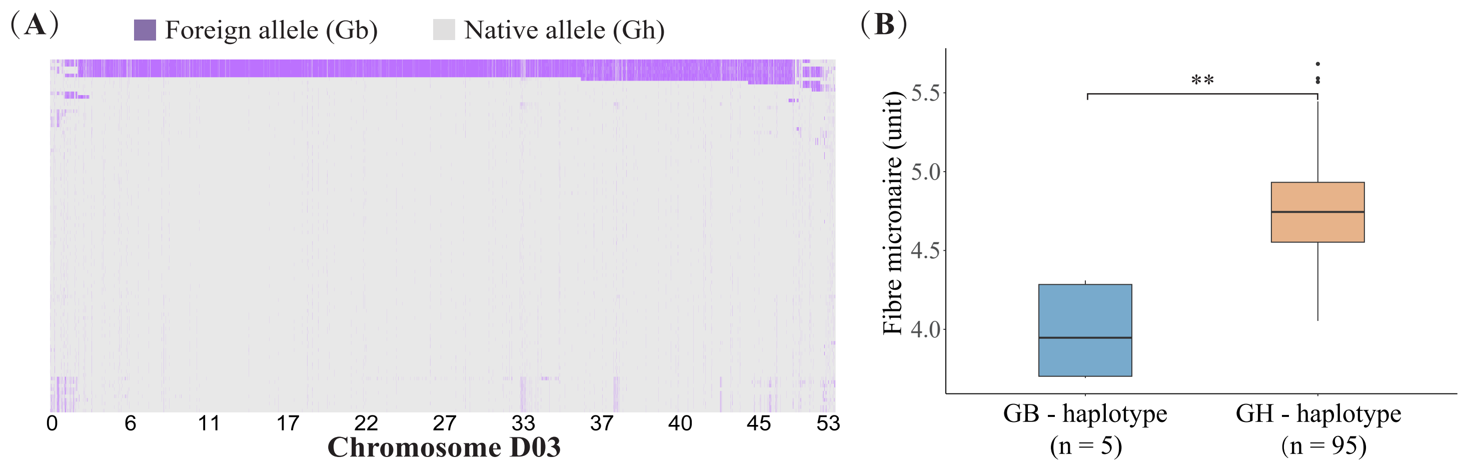

Figure S7. Haplotype analysis for chromosome D03 in the CSSL population. (**A**) Haplotype heatmap for QTL located at chromosome D03. (**B**) Phenotype analysis showed significant differences in fibre micronaire under different D05 QTL haplotypes. Student-t test was applied for the statistic inference.

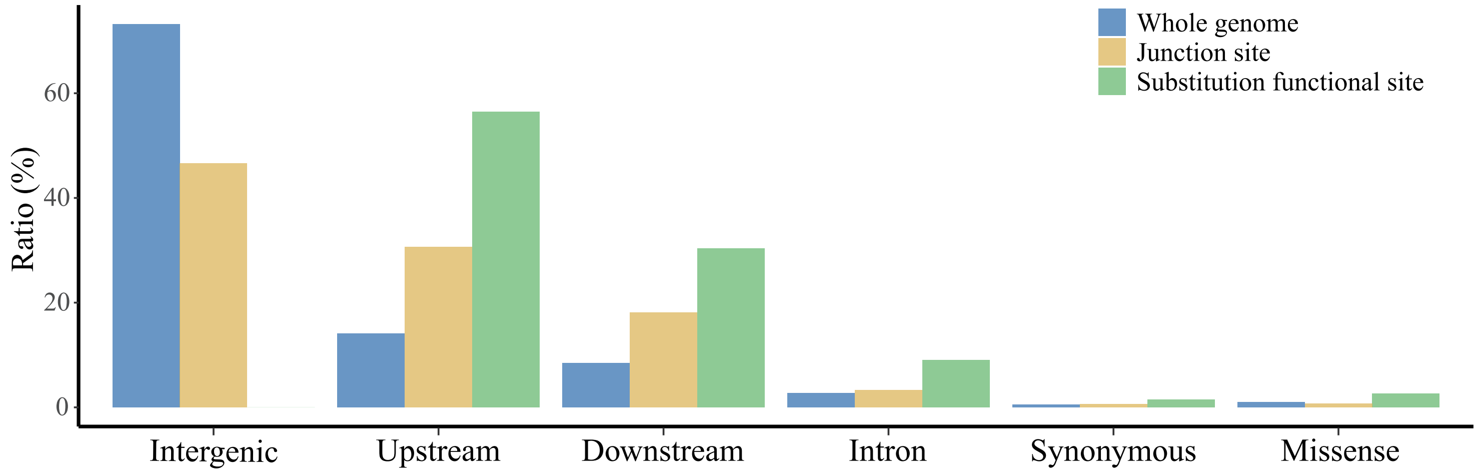

Figure S8. Functional annotation of genomic variants in CSSL population. Whole genome: whole genomic variants. Junction site: variants at the start and end position of all substitution segments. Substitution functional site: variants at gene body, 2,000 bp upstream and downstream of genes that differentially expressed in CSSL, compared to the recurrent parent TM-1.

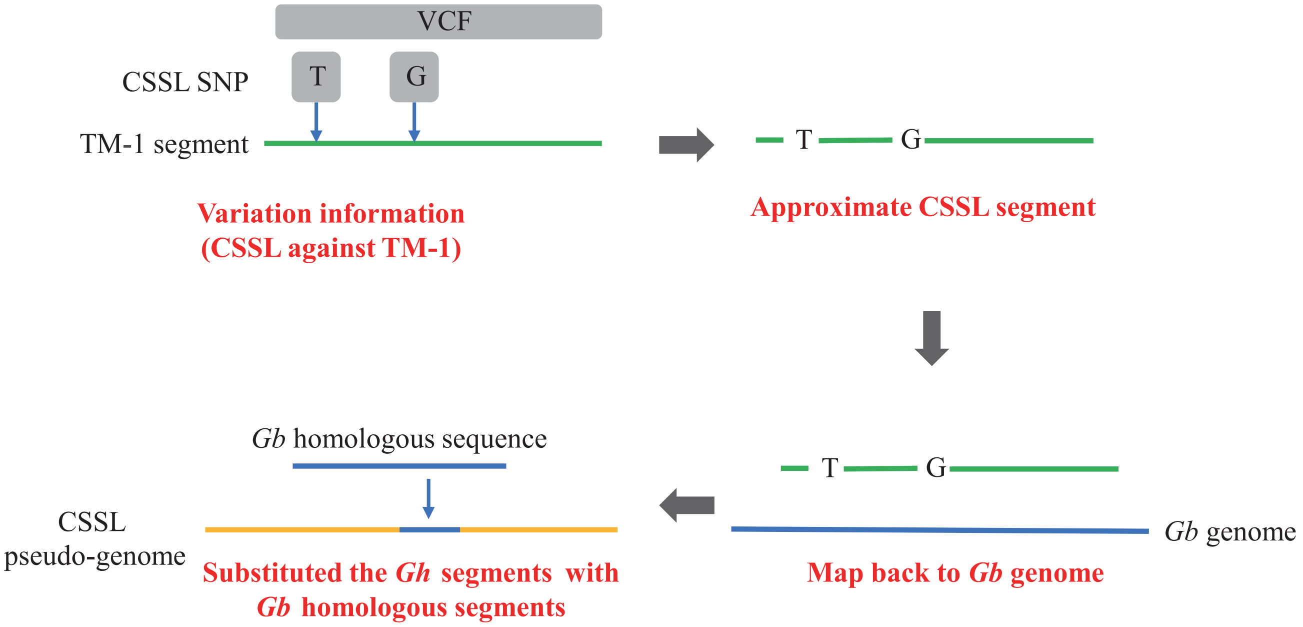

Figure S9. The construction pipeline of CSSL pseudo-genome.

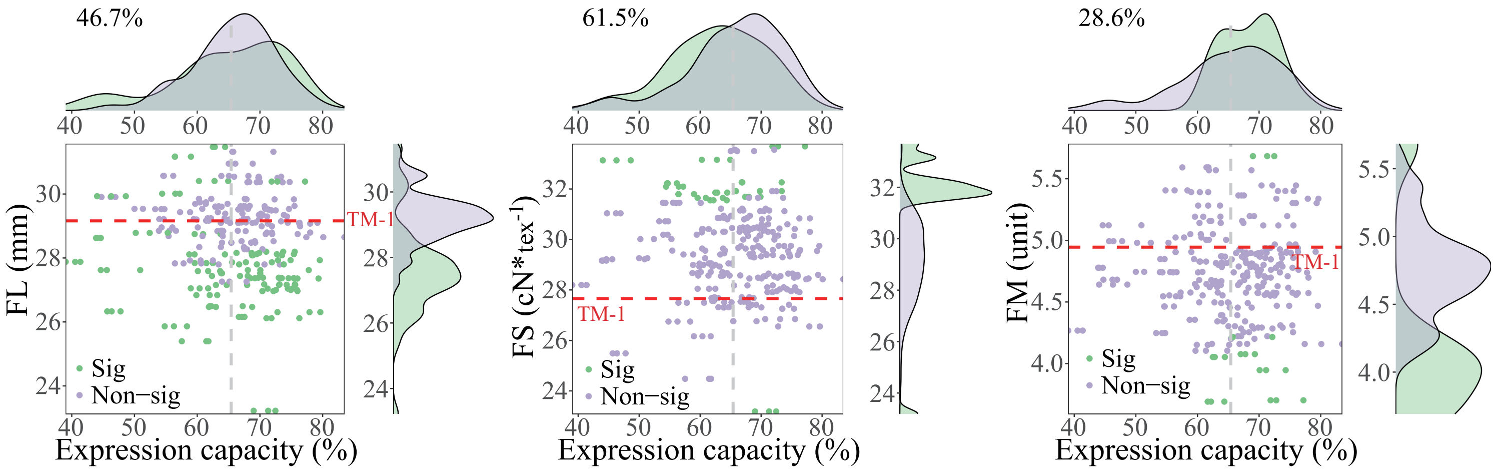

Figure S10. The relationship between expression capacity of foreign introgressed genes across 99 CSSLs and fibre qualities. Introgressed genes have similar distributions of expression capacity regardless whether the CSSL is significantly different from the TM-1 in fibre quality or not. The scatter plot illustrates the expression capacity of the CSSL's fibre transcriptome in three development stages, in conjunction with its associated fibre quality. The CSSL with or without significantly different fibre qualities compared to their recurrent parent TM-1 are marked as green and purple, respectively. The distribution of fibre quality and expression capacity, grouped by whether the CSSL is significantly different from TM-1, are plotted on the side panel and upper panel, respectively. The red dotted line indicates the phenotype of certain fibre quality of TM-1, and grey dotted line notes the dividing point between expression capacity distributions of CSSLs with or without significantly different fibre qualities with TM-1, the number in the upper density plot shows the proportion of CSSLs have divergent fibre quality and simultaneously have incompatible (lower) expression capacities.

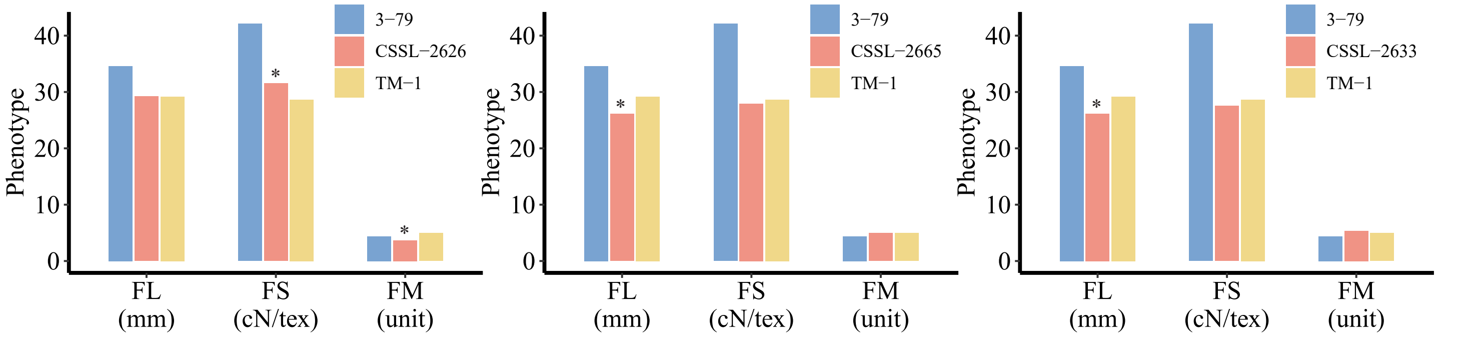

Figure S11. Phenotypic performance of fibre quality for CSSLs with specific genes influenced by foreign introgressed alleles, corrsponds to Figure 3D. Student-t test was applied for the statistical inference. Significant marks were placed above for the phenotypes that significantly differed betweeen CSSL and its recurrent parent TM-1.

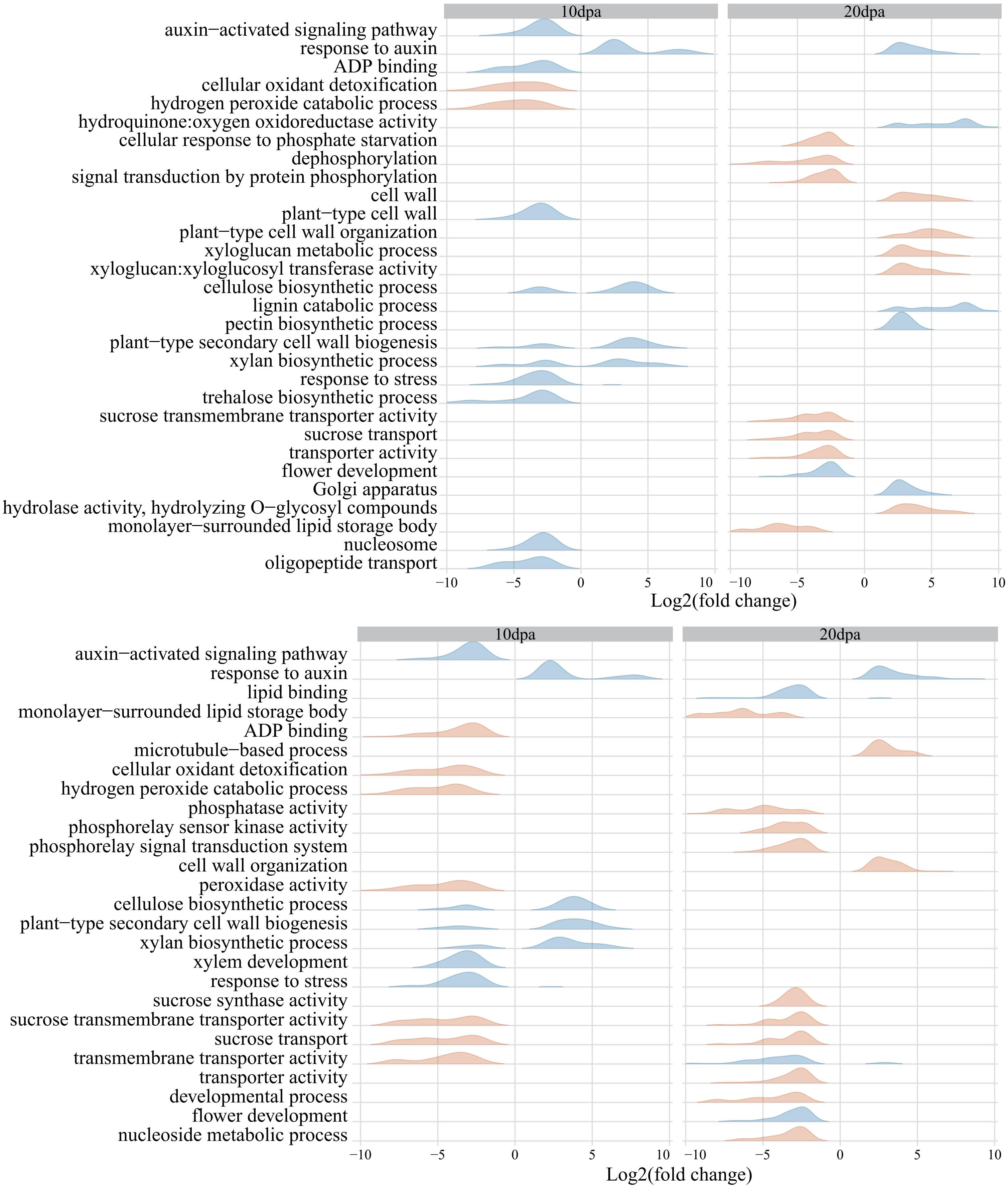

Figure S12. Gene ontology (GO) enrichment pathways of DEGs in CSSLs with superior (orange) or inferior (blue) FS (upper panel) and FM (bottom panel) compared to TM-1 at 10 DPA and 20 DPA. The Y-axis lists GO terms, the X-axis presents the log fold-change value indicating the difference of expression between CSSL and TM-1 for genes enriched in the term, and the ridge height indicates the density of genes with the specific expression pattern.

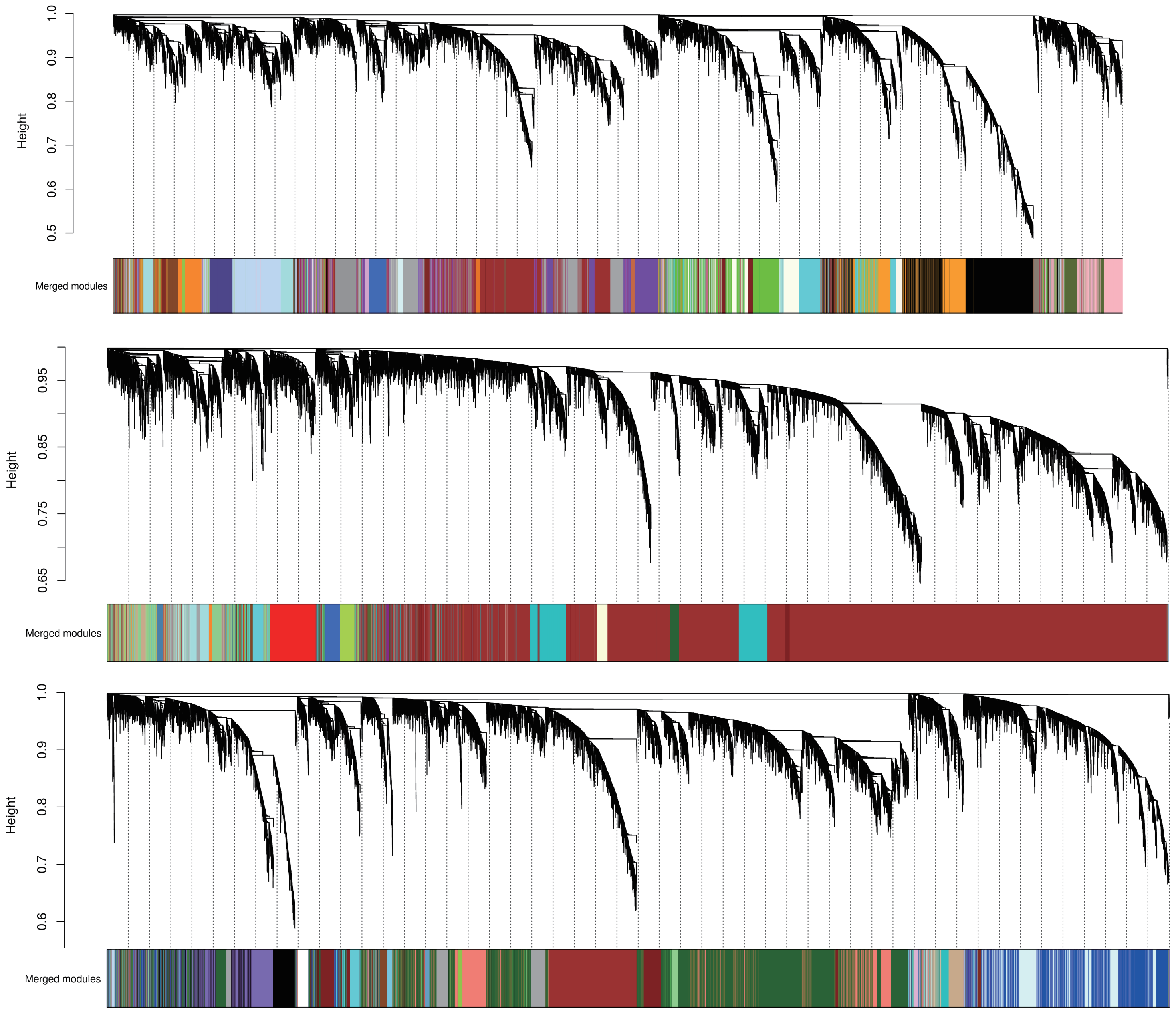

Figure S13. Weighted co-expression regulatory networks constructed for 0 DPA ovlue (up), 10 DPA (middle) and 20 DPA (bottom) fibre. Modules with pair-wised correlation higher than 0.8 were merged, 29, 19 and 21 co-expression modules were identified at last for 0 DPA ovlue and fibres in10 DPA and 20 DPA, respectively.

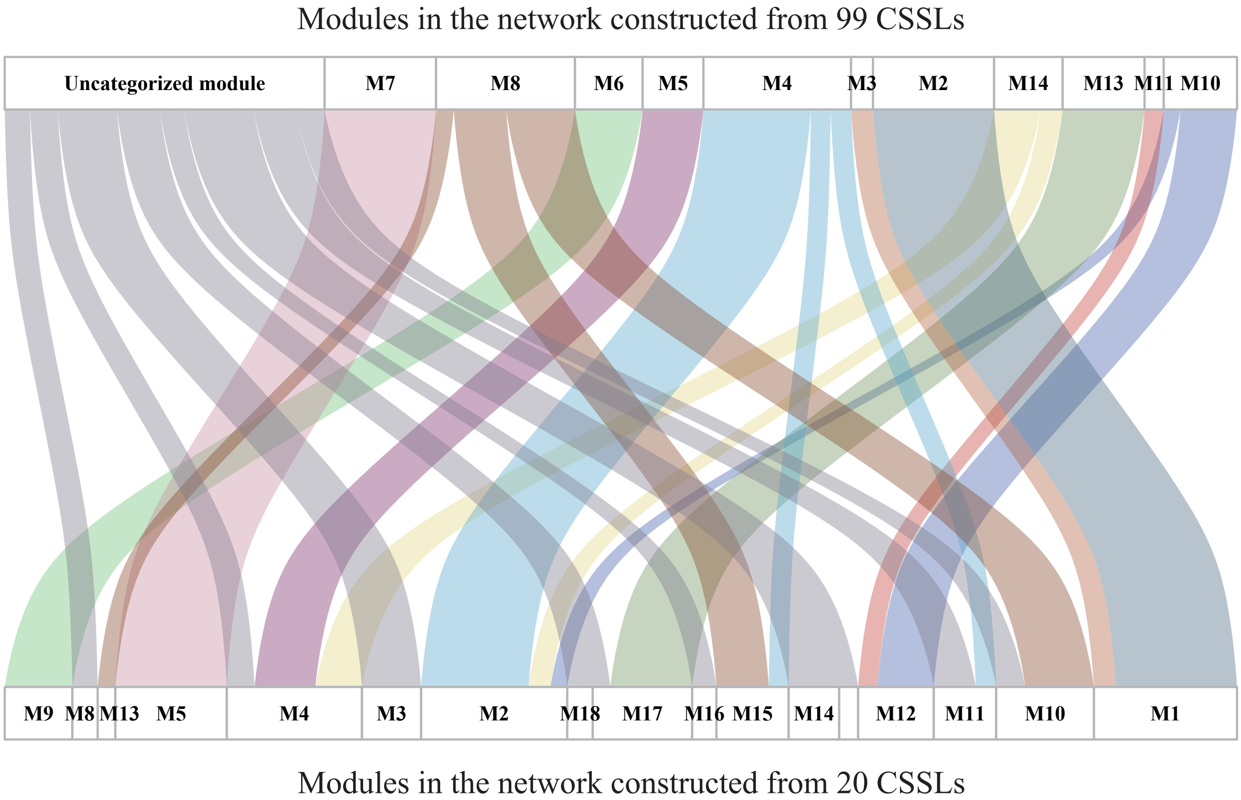

Figure S14. The comparison between co-expressed modules of 10 DPA fibre identified from all 99 CSSLs (up band) and 20 CSSLs (down band) with divergent fibre quality. The uncategorized module in 99-CSSL network were reassigned to valid modules in 20-CSSL network, in which M8, M11,M13 derived from uncategorized module were significantly correlated with the phenotype.

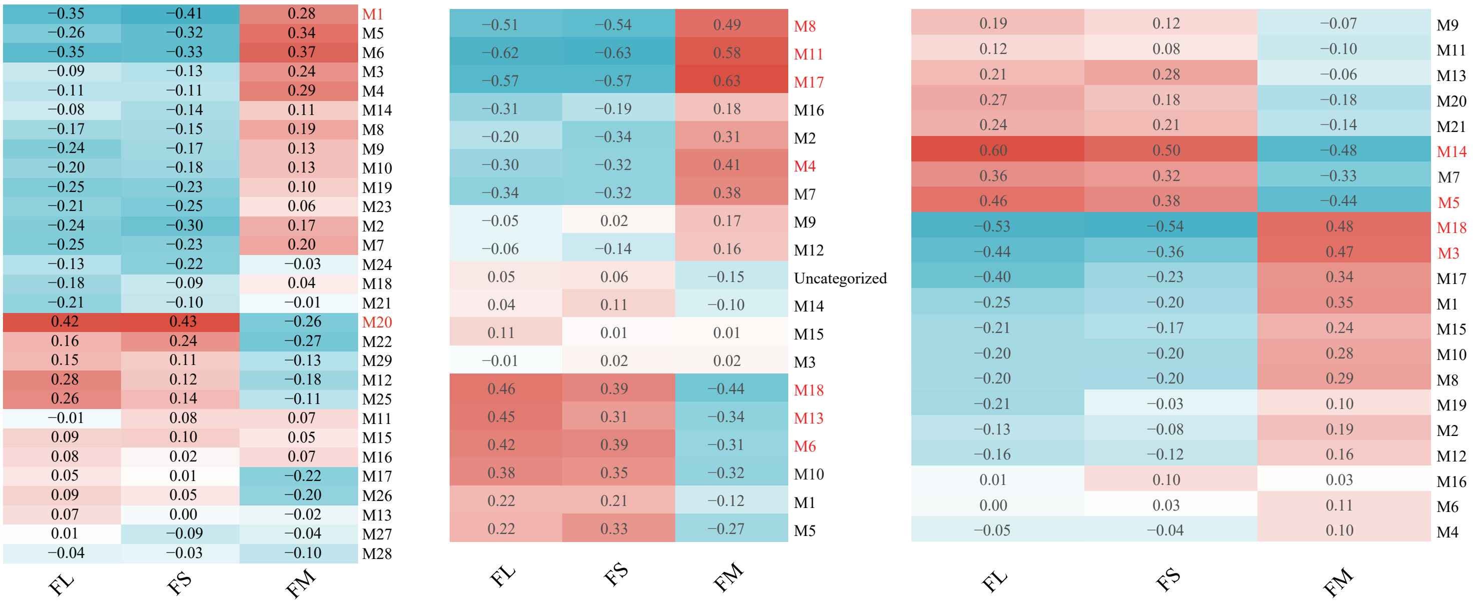

Figure S15. Pearson's correlations between co-expression modules of 0 DPA (left), 10 DPA (middle) and 20 DPA (right) networks and fibre quality traits. The modules with significant correlations (>=0.4) with fibre quality were marked red and used for the network visualization.

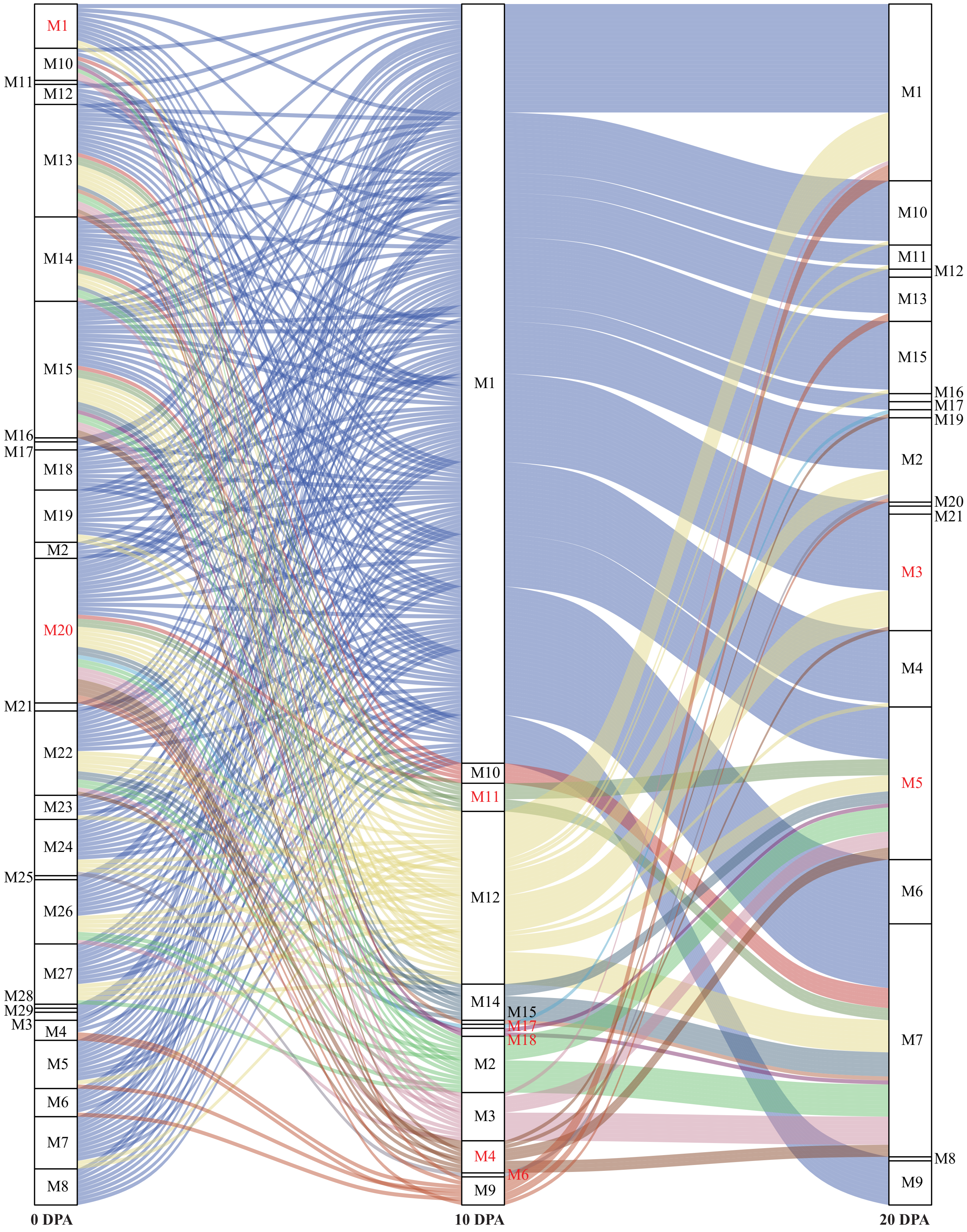

Figure S16. Gene flow patterns of modules in networks constructed from 0 DPA ovlue, 10 DPA and 20 DPA fibre. The co-expression patterns is very different in stages, and expression patterns are more concentrated in 10 DPA fibre. Only data with flow intensity greater than 10 is displayed.

Correspondence of genes in 0 DPA fibre and 10 DPA fibre network

	10dpa M1: 12514	10dpa M2: 860	10dpa M3: 660	10dpa M4: 395	10dpa M5: 65	10dpa M6: 291	10dpa M7: 237	10dpa M8: 272	10dpa M9: 250	10dpa M10: 523	10dpa M11: 404	10dpa M12: 1389	10dpa M13: 63	10dpa M14: 403	10dpa M15: 219	10dpa M16: 122	10dpa M17: 148	10dpa M18: 185	10dpa Uncategorized: 13
0dpa M29: 146	71	7	2	3	0	3	0	0	2	3	1	6	1	2	1	0	2	0	0
0dpa M7: 1444	1073	11	24	7	7	18	20	10	11	16	14	65	0	9	12	8	1	3	0
0dpa M12: 458	221	10	8	5	2	3	6	11	2	7	7	19	1	4	0	0	2	8	0
0dpa M24: 1283	844	17	13	4	1	28	8	18	1	15	16	78	1	8	5	4	5	3	1
0dpa M20: 2501	1063	122	76	153	3	19	21	25	48	42	34	143	2	48	23	17	42	13	0
0dpa M26: 1014	501	51	22	5	2	12	12	12	7	26	23	90	8	8	22	8	4	10	0
0dpa M22: 1470	693	98	52	30	2	19	13	20	13	31	28	129	3	31	18	8	17	13	0
0dpa M15: 3076	1524	131	111	45	6	37	26	27	31	83	53	152	3	63	34	17	15	27	2
0dpa M27: 1133	583	32	19	7	1	19	10	10	7	16	15	80	14	11	5	3	4	9	1
0dpa M1: 519	395	4	5	4	2	8	13	2	1	5	4	44	2	2	1	1	0	1	0
0dpa M14: 1435	843	49	35	20	2	19	14	19	5	35	19	85	4	32	21	8	9	14	0
0dpa M5: 692	483	15	6	2	1	7	5	5	18	9	6	37	1	7	2	4	0	2	0
0dpa M10: 387	84	31	46	15	0	11	3	4	4	56	11	12	2	29	12	4	1	23	0
0dpa M18: 532	340	16	13	2	10	6	7	5	2	9	3	24	0	8	1	2	3	2	1
0dpa M8: 506	346	8	12	2	0	6	5	6	2	15	7	23	0	10	0	6	0	4	0
0dpa M28: 112	45	12	6	8	0	0	2	0	0	1	3	9	0	8	1	0	0	4	0
0dpa M2: 238	163	4	2	0	0	2	4	1	0	5	1	16	0	2	2	2	1	0	1
0dpa M16: 121	72	3	2	1	0	2	2	1	0	0	2	7	0	2	2	1	0	2	0
0dpa M13: 2024	1065	110	52	26	0	24	18	25	26	45	44	137	11	35	23	3	11	14	0
0dpa M19: 962	636	22	8	4	21	19	15	10	2	11	6	77	1	6	2	2	5	2	0
0dpa M23: 448	287	10	5	0	1	12	1	2	3	0	2	47	1	1	3	0	0	1	1
0dpa M25: 61	34	6	0	0	0	0	2	0	0	0	1	4	0	0	0	0	0	0	0
0dpa M21: 130	84	4	4	2	0	2	0	4	0	2	0	14	0	0	1	1	1	0	0
0dpa M4: 322	212	3	6	0	0	3	3	3	35	3	1	20	1	2	0	1	0	0	0
0dpa M9: 91	27	2	10	2	0	2	0	0	0	11	4	10	0	3	0	2	0	4	1
0dpa M6: 237	173	0	5	0	0	0	0	0	15	1	2	6	1	4	0	1	1	1	0
0dpa M11: 67	29	4	2	0	0	0	0	0	0	0	3	6	1	1	1	0	3	0	0
0dpa M3: 192	143	4	3	0	0	1	1	0	4	1	2	9	0	1	1	0	0	0	0
0dpa M17: 110	83	2	3	0	0	1	0	1	0	4	0	2	0	1	0	1	1	1	0

$-\log_{10}(p)$: 0, 10, 20, 30, 40, 50

Figure S17. The correspondence of genes in the networks contrcuted from 0 DPA and 10 DPA transcriptomic data. The x-axis and y-axis are labelled with the timepoint, the modules and the total number of genes contained in it, and modules that are significantly correlated with the fibre quality traits were marked red. The color blocks along the axis are the corresponding colors in network diagram as shown in Figure 6 (if present). The numbers in the matrix are overlapped genes between pair-wised modules, and their colors represent the significant level of consensus detection, by applying fisher-exact test.

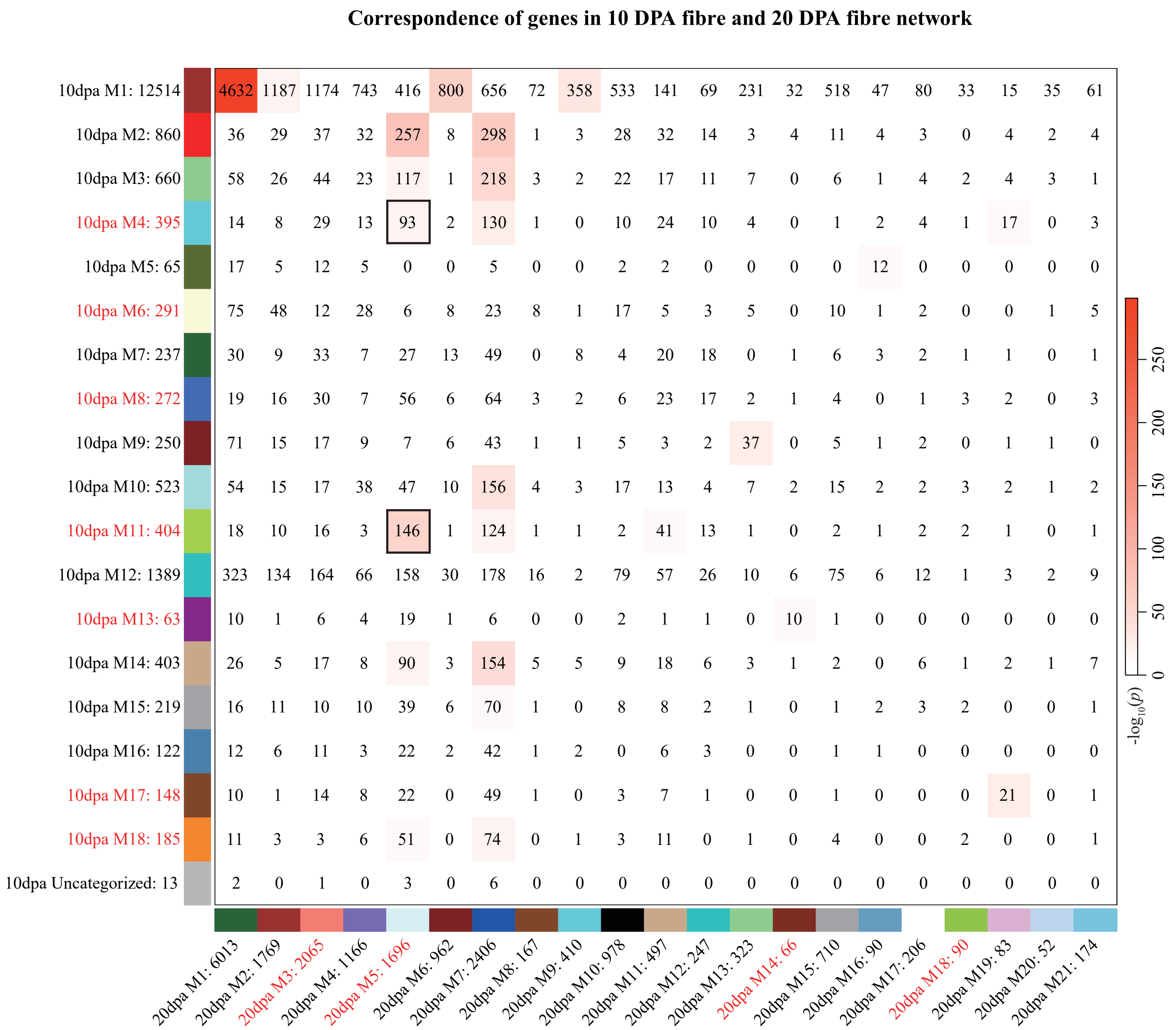

Figure S18. The correspondence of genes in the networks contrcuted from 10 DPA and 20 DPA transcriptomic data. The x-axis and y-axis are labelled with the timepoint, the modules and the total number of genes contained in it, and modules that are significantly correlated with the fibre quality traits were marked red. The color blocks along the axis are the corresponding colors in network diagram as shown in Figure 6 (if present). The numbers in the matrix are overlapped genes between pair-wised modules, and their colors represent the significant level of consensus detection, by applying fisher-exact test.

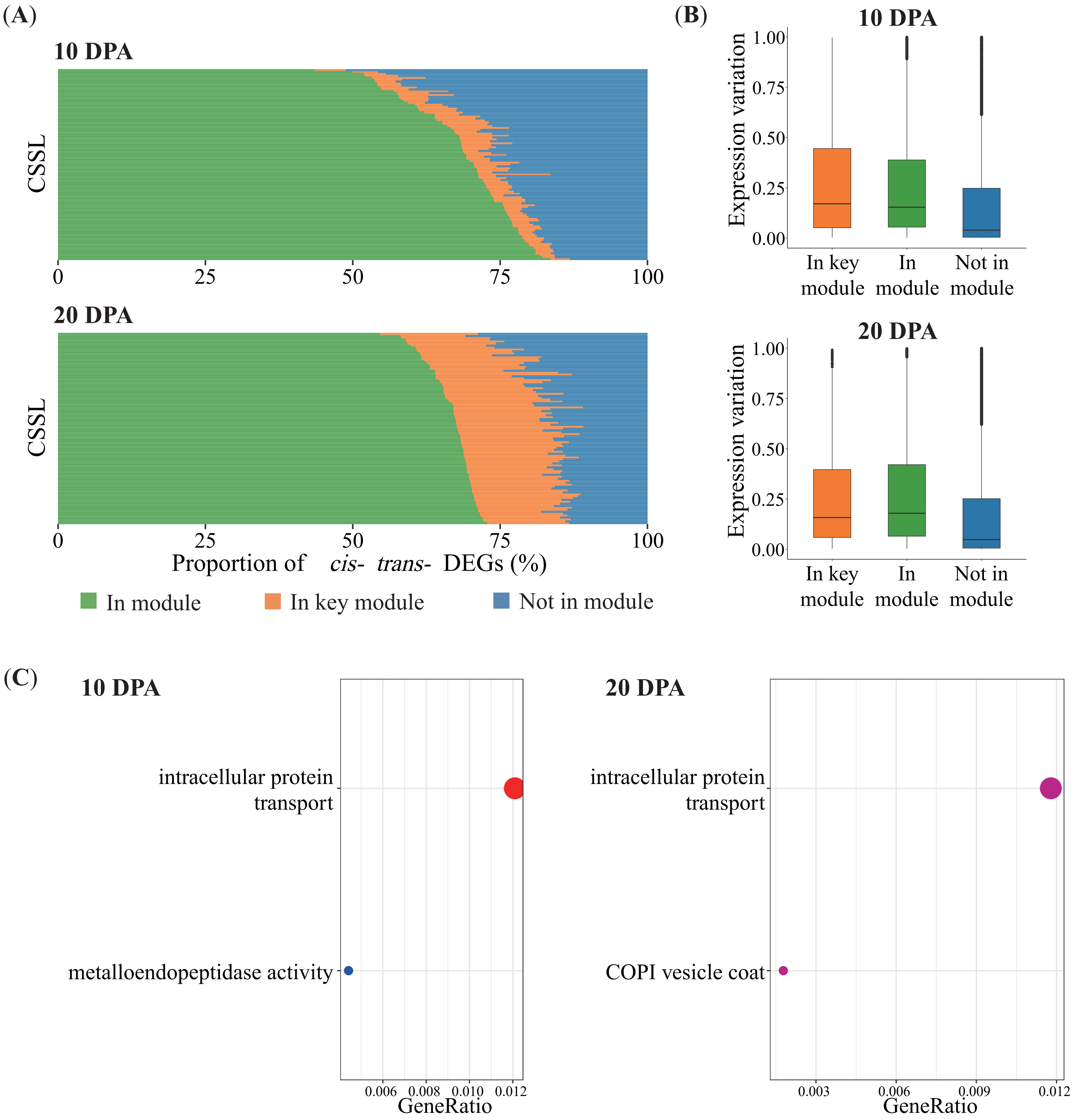

Figure S19. *Cis*- and *trans*-DEGs included in the co-expression regulatory network. (**A**) The proportion of *cis*- and *trans*-DEGs included in the networks of 10 DPA and 20 DPA fibre. (**B**) The variance of expression level across the CSSL population for genes assigned with different status. (**C**) Enrichment analysis exploring the potential biological functions for genes not included in any of the co-expression modules in 10 DPA and 20 DPA regulatory networks.

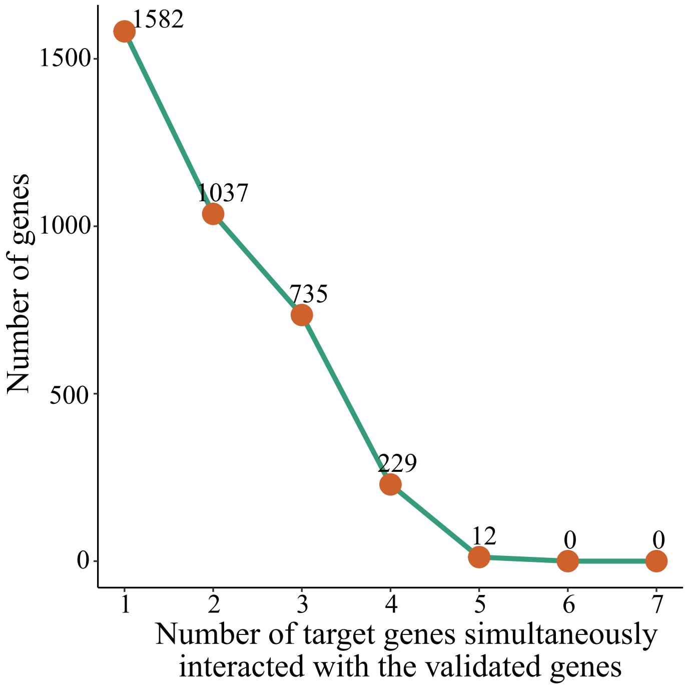

Figure S20. Number of genes that simultaneously interacted with validate genes in 20 DPA co-expression regulatory network. A total of 12 genes that simultaneously interacted with 5 validate genes were predicted to be functional in fibre improvement.

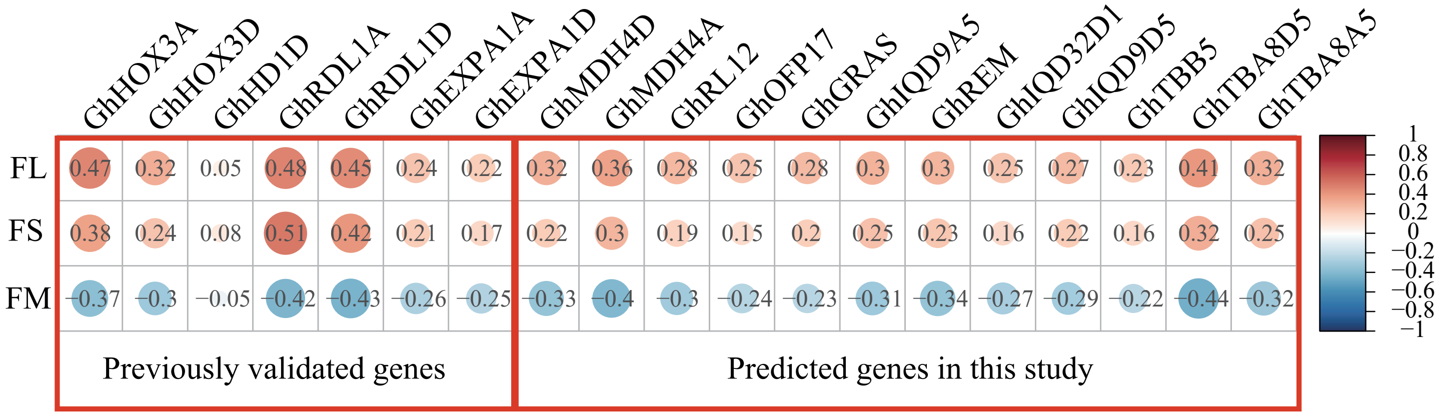

Figure S21. Pearson correlation between the expression levels of the 19 genes of interests and fibre qualities.

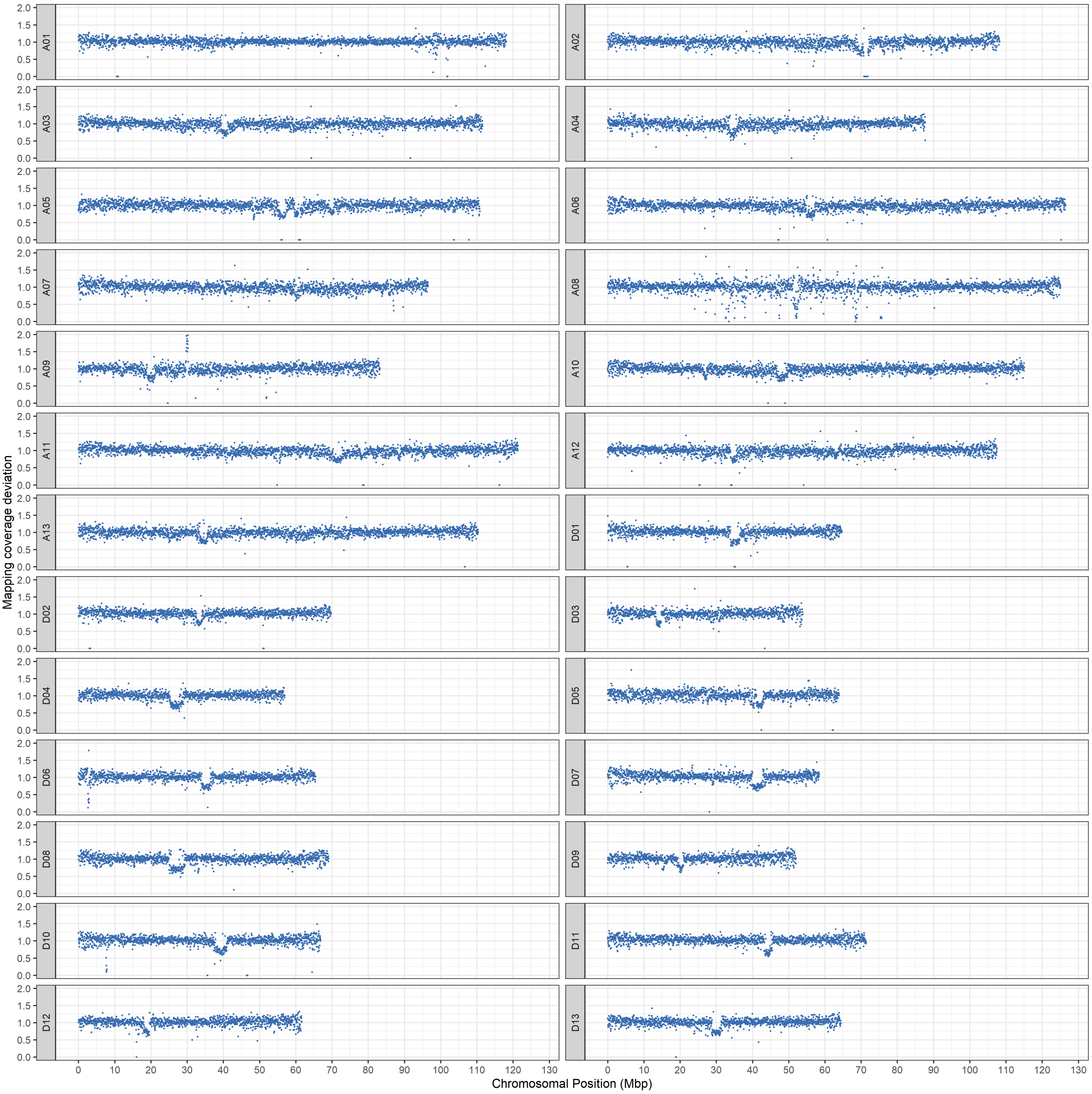

Figure S22. Detecing substitution segments in CSSL-2656 using coverage-based method as described by Coombes *et al.*. The results show no obvious regions with sharp decreased coverage in the CSSL, but a region with slightly lower coverage on every chromosome, which indicated to be centromere of cotton TM-1.
