# Supplementary material for: Unravelling the genetic basis and regulation networks related to fibre quality improvement using chromosome segment substitution lines in cotton
Source: Plant Biotechnol J. 2024 Jul 24;22(11):3135–50. doi: 10.1111/pbi.14436 (PMC11500987; doi:10.1111/pbi.14436)
Supplement: Supplementary file 3 — Appendix S1 Supporting Methods. [file PBI-22-3135-s001.docx]

Supporting Methods

**Unraveling the genetic basis and regulation networks related to fibre quality improvement using chromosome segment substitution lines in cotton**

Guoan Qi^1,2^, Zhanfeng Si^2^, Lisha Xuan^2^, Zegang Han^2^, Yan Hu^1, 2^, Lei Fang^1, 2^, Fan Dai^2^, Tianzhen Zhang^1,2,*^

**Correspondence:** *Tianzhen Zhang (cotton@zju.edu.cn)

**Supporting Methods**

**Cultivation period and location of the CSSL population.** The 174 Hai7124 CSSLs were cultivated from year 2001 to 2008 at Nanjing (Eastern China, temperate) in summer and at Sanya (Southern China, tropical) in winter (Wang et al., 2008; Wang et al., 2012). The 166 3-79 CSSLs constructed in the current study were cultivated from 2010 to 2016 at Nanjing or Dangtu (Eastern China, temperate) in the summer and at Sanya in the winter.

In 2015 and 2016, we planted the 166 3-79 CSSLs in Nanjing, Jiangsu province and Dangtu, Anhui province, China. Field performance was assessed in terms of yield and fibre qualities. Likewise, the 174 G. barbadense acc. Hai7124 CSSLs were grown from year 2010 to 2016 at Nanjing or Dangtu in summer and at Sanya in winter. From these, we selected that differed significantly from TM-1 in either lint yield or fibre quality. In total, 99 CSSLs were selected for the present study.

**Detailed procedures for the detection of substitution segments.** The detection strategy comprised three main steps: allele classification, slide window analysis, and ambiguous allele adjustment.

**Allele classification:** Comparison of genotypes between a CSSL and its parental lines allows the parental origin of alleles to be determined. All alleles can be assigned to three groups: lineage-specific alleles (LSAs), non-lineage-specific alleles (NLSAs), and redundant alleles (RAs). LSAs are those alleles with clear parental origin; for example, considering a locus where the respective genotypes in the donor and recurrent parental lines are AA and aa, and the genotype in the CSSL is aa, then the offspring clearly inherited that locus from the recurrent line. Similarly, we can also identify loci of donor line origin. NLSAs are those alleles with ambiguous origin; a straightforward example is a locus whose genotypes in the CSSL and its parental lines are all heterozygous, in which case the parental origins of the CSSL alleles cannot be determined. RAs are those alleles without polymorphisms or for which genotype is missing in either parent or the offspring line. As these alleles provide barely any useful information in the analysis, they were removed in our pipeline.

**Slide window analysis:** In the previous step, we identified the parental origin of genomic SNPs, and the draft distribution of allele types across the genome allowed the preliminary localization of substitution segments. To further localize the substitution segment and reduce false positives caused by confounding factors such as sequencing error, we calculated the local substitution proportion (LSP) by dividing the number of LSAs from the donor line by the total number of loci in a given genomic window. A window is assigned to a substitution segment if the LSP is higher than 80%. This step is similar in principle with identifying SNP bins based on the population-level crosspoints: the SNP binner predicts the genotype region based on the homogeneity of the alternative haplotype (Gonda el al., 2019), whereas our method identifies foreign introgression regions based on the homogeneity of donor parent-specific haplotype.

**Ambiguous allele adjustment:** It is usual that a large number of NLSAs are detected. These loci have not been used for substitution segment detection to date, which could negatively impact detection sensitivity. We propose that the origin of a NLSA can be inferred from LD based on the clear origins of its neighboring alleles, if available. Thus, in addition to donor line LSAs, we also considered NLSAs in each genomic window and their abundance relative to LSAs. If most of the alleles in a given genomic window are donor line LSAs and NLSAs, and none or only very few are recurrent line LSAs, then the NLSAs would be reclassified as donor line LSAs. By default, this correction is triggered when the LSP (calculated in the previous step) in a genomic region is at least five times greater than the proportion of native LSAs. Genomic regions for which the updated LSP exceeds 80% are considered to belong to a substitution segment.

DNA sequencing and variant calling. DNA was extracted from the young leaves of CSSLs using the standard CTAB method and sequenced on the Illumina 10X Genomics platform. About 739 gigabytes of paired-end reads with 150 bp insert size were generated. We used FASTP to conduct quality control on the raw sequencing data, trimming adapter bases, bases with quality value lower than 15, reads with unqualified bases over 40%, and reads with length shorter than 15 (Chen et al., 2018). The resulting high-quality reads were aligned to the *G. hirsutum* acc. TM-1 reference genome using BWA, PCR duplicates were marked using Sambamba, and finally genetic variation was called using the bcftools pipeline (Hu et al., 2019; Li, 2011; Li and Durbin, 2009; Tarasov et al., 2015). For quality control, any variants with a confidence score (QUAL) lower than 30 were filtered out from the raw calling result.

RNA sequencing and transcriptome quantification. RNA was extracted from 0 DPA ovule and 10 and 20 DPA fibre of the CSSLs and their parental lines, please refer to Table S19 for detailed information for the samples. A total of ~2.64 terabytes of paired-end sequencing data were generated on the Illumina NovaSeq 6000 platform. Raw data was pre-processed by FASTP with the same parameters as applied to DNA raw sequencing data. The clean data was mapped against the *G. hirsutum* acc. TM-1 reference genome to obtain a unified transcriptomic panel, and against the pseudo-genome of the corresponding CSSL (detailed below) using hisat2; reads with exact alignment were retained for subsequent analysis (Kim et al., 2019). Transcriptome quantification was performed using Stringtie and HTseq-count (Putri et al., 2022; Shumate et al., 2022); HTseq-count was employed in union mode, and reads with mapping quality lower than 10 were ignored. Orthologues between TM-1, 3-79, and Hai7124 were identified using OrthoFinder2 with default parameters (Emms and Kelly, 2019).

Association study. The genome-wide association studies for fibre quality traits were conducted with PLINK2 with --bfile specifies the genotype matrix, --covar specifies the covariates included, --pheno specifies the phenotypes, and additional parameters “--allow-extra-chr –glm hide-covars” (Chang et al., 2015). SNP markers with minor allele frequency lower than 0.05 or missing rate higher than 0.2 were filtered out; after this quality control, 3,263,105 SNP markers remained for the association analysis. The best linear unbiased estimator of fibre quality traits was applied as the response variable, and the top two eigenvectors representing possible population stratification were included as covariates in the linear regression model. SNP makers were defined as significant if the association *P*-value was lower than the suggested threshold of 1e-5. For significant SNP markers, two genetic models were constructed, with or without consideration of the test marker, for the calculation of phenotypic variance explained (PVE). Both models used fibre quality phenotype values as response variables and the first two eigenvectors as covariates. PVE was calculated as the difference in the linear goodness of fit between the models.

Analysis of differentially expressed genes and regulatory pathways. We undertook a comprehensive analysis of the differentially expressed genes (DEG) and regulatory pathways associated with FL, FS, and FM. We selected five CSSLs with superior fibre quality and five with inferior fibre quality, then determined differential expression of genes between those CSSLs and their recurrent parental line TM-1 following the DESeq2 pipeline; raw counts of genes based on the TM-1 reference genome were used here. Transcripts with absolute log2 fold-change higher than 2 and adjusted *P*-value lower than 0.05 were defined as DEGs. The R package *clusterProfiler* was utilized for Gene Ontology (GO) enrichment analysis (Yu et al., 2012). A GO term was defined as differentially expressed if multiple genes annotated with the term were significantly differentially expressed in the CSSLs.

Construction of co-expression regulation networks. Network analysis was applied to a joint gene set comprising 23,384 DEGs, identified in the context of TM-1 reference genome, across three key fibre developmental stages. Transcripts with low variation were removed from the set. We constructed the scale-free co-expression network using the R package *WGCNA* (Langfelder and Horvath, 2012). Soft thresholds were chosen to guarantee the fitness of scale-free network as greater than 0.8. For the modules detected, we merged similar modules if their pairwise correlation was greater than 0.8. The first eigenvector of each module was calculated as an eigengene and regressed against the fibre quality phenotypes to determine the correlation and significance. Kernel modules are those having absolute pairwise correlation greater than 0.4 and a regression *p*-value lower than 0.05.

Yeast two-hybrid assay (Y2H). To validate protein interactors from the co-expression network, the coding sequences of *GhHOX3* and *GhTBA8A5* were amplified and cloned into the pGADT7 vector, and the *GhRDL1* coding region was cloned into the pGBKT7 vector. The vectors were co-transformed into Y2H competent yeast cells, with positive clones confirmed by PCR amplification analysis. Interactions were detected by serial dilution (1:10) and spotting on SD/-Trp-Leu and SD/-Trp-Leu-His media.

**SI References**

Chang, C.C., Chow, C.C., Tellier, L.C., Vattikuti, S., Purcell, S.M. and Lee, J.J. (2015) Second-generation PLINK: rising to the challenge of larger and richer datasets. *GigaScience* **4**, s13742-015-0047-8.

Chen, S., Zhou, Y., Chen, Y. and Gu, J. (2018) fastp: an ultra-fast all-in-one FASTQ preprocessor. *Bioinformatics* **34**, i884-i890.

Emms, D.M. and Kelly, S. (2019) OrthoFinder: phylogenetic orthology inference for comparative genomics. *Genome Biology* **20**, 238.

Gonda, I., Ashrafi, H., Lyon, D.A., Strickler, S.R., Hulse-Kemp, A.M., Ma, Q., Sun, H., Stoffel, K., Powell, A.F., Futrell, S., Thannhauser, T.W., Fei, Z., Van Deynze, A.E., Mueller, L.A., Giovannoni, J.J. and Foolad, M.R. (2019) Sequencing-based bin map construction of a tomato mapping population, facilitating high-resolution quantitative trait loci detection. *The Plant Genome*, 12: 180010.

Hu, Y., Chen, J., Fang, L., Zhang, Z., Ma, W., Niu, Y., Ju, L., Deng, J., Zhao, T., Lian, J., Baruch, K., Fang, D., Liu, X., Ruan, Y.-l., Rahman, M.-u., Han, J., Wang, K., Wang, Q., Wu, H., Mei, G., Zang, Y., Han, Z., Xu, C., Shen, W., Yang, D., Si, Z., Dai, F., Zou, L., Huang, F., Bai, Y., Zhang, Y., Brodt, A., Ben-Hamo, H., Zhu, X., Zhou, B., Guan, X., Zhu, S., Chen, X. and Zhang, T. (2019) *Gossypium barbadense* and *Gossypium hirsutum* genomes provide insights into the origin and evolution of allotetraploid cotton. *Nature Genetics* **51**, 739-748.

Kim, D., Paggi, J.M., Park, C., Bennett, C. and Salzberg, S.L. (2019) Graph-based genome alignment and genotyping with HISAT2 and HISAT-genotype. *Nature Biotechnology* **37**, 907-915.

Langfelder, P. and Horvath, S. (2012) Fast R Functions for Robust Correlations and Hierarchical Clustering. *Journal of Statistical Software* **46**, 1-17.

Li, H. (2011) A statistical framework for SNP calling, mutation discovery, association mapping and population genetical parameter estimation from sequencing data. *Bioinformatics* **27**, 2987-2993.

Li, H. and Durbin, R. (2009) Fast and accurate short read alignment with Burrows–Wheeler transform. *Bioinformatics* **25**, 1754-1760.

Putri, G.H., Anders, S., Pyl, P.T., Pimanda, J.E. and Zanini, F. (2022) Analysing high-throughput sequencing data in Python with HTSeq 2.0. *Bioinformatics* **38**, 2943-2945.

Shumate, A., Wong, B., Pertea, G. and Pertea, M. (2022) Improved transcriptome assembly using a hybrid of long and short reads with StringTie. *PLOS Computational Biology* **18**, e1009730.

Tarasov, A., Vilella, A.J., Cuppen, E., Nijman, I.J. and Prins, P. (2015) Sambamba: fast processing of NGS alignment formats. *Bioinformatics* **31**, 2032-2034.

Wang, P., Ding, Y., Lu, Q., Guo, W. and Zhang, T. (2008) Development of *Gossypium barbadense* chromosome segment substitution lines in the genetic standard line TM-1 of *Gossypium hirsutum*. *Chinese Science Bulletin* **53**, 1512-1517.

Wang, P., Zhu, Y., Song, X., Cao, Z., Ding, Y., Liu, B., Zhu, X., Wang, S., Guo, W. and Zhang, T. (2012) Inheritance of long staple fibre quality traits of *Gossypium barbadense* in *G. hirsutum* background using CSILs. *Theoretical and Applied Genetics* **124**, 1415-1428.

Yu, G., Wang, L.-G., Han, Y. and He, Q.-Y. (2012) clusterProfiler: an R Package for Comparing Biological Themes Among Gene Clusters. *OMICS: A Journal of Integrative Biology* **16**, 284-287.
